# Priorities for Standards and Measurements to Accelerate Innovations in Nano-Electrotechnologies: Analysis of the NIST-Energetics-IEC TC 113 Survey+,*

**DOI:** 10.6028/jres.114.008

**Published:** 2009-04-01

**Authors:** Herbert S. Bennett, Howard Andres, Joan Pellegrino, Winnie Kwok, Norbert Fabricius, J. Thomas Chapin

**Affiliations:** Semiconductor Electronics Division, National Institute of Standards and Technology, Gaithersburg, MD 20899, USA; Energetics Incorporated, Columbia, MD 21046, USA; Forschungszentrum Karlsruhe GmbH, Eggenstein-Leoppoldshafen, D-76344 Germany; Underwriters Laboratories Inc., Northbrook, IL 60062, USA

**Keywords:** Borda count method, confidence interval, median method, nano-electrotechnologies, priorities, rankings, standards, statistical significance

## Abstract

In 2008, the National Institute of Standards and Technology and Energetics Incorporated collaborated with the International Electrotechnical Commission Technical Committee 113 (IEC TC 113) on nano-electrotechnologies to survey members of the international nanotechnologies community about priorities for standards and measurements to accelerate innovations in nano-electrotechnologies. In this paper, we analyze the 459 survey responses from 45 countries as one means to begin building a consensus on a framework leading to nano-electrotechnologies standards development by standards organizations and national measurement institutes. The distributions of priority rankings from all 459 respondents are such that there are perceived distinctions with statistical confidence between the relative international priorities for the several items ranked in each of the following five Survey category types: 1) Nano-electrotechnology Properties, 2) Nano-electrotechnology Taxonomy: Products, 3) Nano-electrotechnology Taxonomy: Cross-Cutting Technologies, 4) IEC General Discipline Areas, and 5) Stages of the Linear Economic Model. The global consensus prioritizations for ranked items in the above five category types suggest that the IEC TC 113 should focus initially on standards and measurements for electronic and electrical properties of sensors and fabrication tools that support performance assessments of nano-technology enabled sub-assemblies used in energy, medical, and computer products.

## 1. Introduction

In this paper, we present the results from a recent international Survey to establish priorities for standards and measurements involving nano-electrotechnologies. We describe the origin and compelling reasons for conducting the survey; the survey structure and its online distribution; the demographics of survey respondents; an analysis of the ranking data obtained from the Survey; and the major findings. The Survey included all stages of the economic cycle for nano-electrotechnology enabled products and systems from research to end-of-useful life, disposal, and/or recycling.

Sections 2 and 3 present the background, origin, structure, methodology, and demographics for the Survey. Section 4 contains the statistical details for the ranking priorities. Section 5 gives the results of selected pair-wise correlations. Section 6 contains a summary of just the major results and serves as an executive summary without statistical details. Appendix A contains a copy of the Survey as it appeared on the website. And finally, Appendix B discusses the statistics and formulas on which we base our findings and results from the Survey.

### 1.1 Nanotechnology Defined

There are many definitions of nanotechnology. The definition from the U.S. National Nanotechnology Initiative encompasses key aspects included in other definitions from around the world. “*Nanotechnology is the understanding and control of matter at dimensions between approximately 1 and 100 nanometers, where unique phenomena enable novel applications. Encompassing nanoscale science, engineering, and technology, nanotechnology involves imaging, measuring, modeling, and manipulating matter at this length scale.* …. *Dimensions between approximately 1 and 100 nanometers are known as the nanoscale. Unusual physical, chemical, and biological properties can emerge in materials at the nanoscale. These properties may differ in important ways from the properties of bulk materials and single atoms or molecules.*” [1]

Nano-electrotechnologies are part of nanotechnology. They are often cross-sectional technologies with the potential for many cross-disciplinary applications. From the perspective of the International Electrotechnical Commission (IEC), nano-electrotechnologies [2] include the following areas at the nanoscale: nanostructured sensors; nano-electronics, nano-materials and nano-devices; optoelectronics; optical materials and devices; organic (opto)-electronics; magnetic materials and devices; radio frequency devices, components and systems; electrodes with nanostructured surfaces; electrotechnical properties of nanotubes/nanowires; analytical equipment and techniques for measurement of electrotechnical properties; patterning equipment and techniques; masks and lithography; performance, durability, and reliability assessment for nanoelectronics; fuel cells; and bio-electronic applications.

### 1.2 The Standards and Innovation Connection

Nano-electrotechnologies are expected to be one of the key technologies of the 21st century and to provide enormous potential for the development of new products with exceptional performance. Nano-electrotechnologies will enable society to take advantage of economic successes as well as improvements in the quality of life by using nano-enabled products. One example in healthcare is wireless monitoring of health and safety in an aging society, especially for assisted living in the home or in facilities. Reliability and durability of nano-enabled medical products are great challenges because the mainstream nanoelectronics industry now often favors performance at the expense of reliability and durability [3].

International commerce in nano-electrotechnologies will require technically valid standards and related measurements that are suitable for use in any nation. These standards must therefore be developed with input from all stakeholders. Effective international standards will facilitate wider use of products that offer greater functionality or performance through nano-electrotechnologies-enabled subassemblies. They will also enhance the health and safety aspects of products for the protection of researchers, manufacturers, consumers, and the environment.

According to a recently published report of Semiconductor Equipment and Materials International (SEMI) in cooperation with the Semiconductor Industry Association (SIA) [4] and by the RNCOS Group [5], the materials and equipment market for nanoelectronics was US $1.8 billion in 2005 and is expected to be US $4.2 billion in 2010. The semiconductor electronics industry is already a nanotechnology industry and will be increasingly important in the future. The continued rapid growth of this and other nano-electrotechnologies-based industries has required increased international standardization activities to support equitable and efficient business models.

### 1.3 Role of IEC Technical Committee 113 on Nano-Electrotechnologies

Given the importance of standards to this emerging field, the Standardization Management Board of the International Electrotechnical Commission (IEC SMB) established an Advisory Board on Nanotechnologies (SMB ABN 20) in 2005. Based on the recommendations from the members of ABN 20, the IEC SMB established in May 2006 the IEC Technical Committee 113 (IEC TC 113) on Nanotechnology Standardization for Electrical and Electronic Products and Systems [6]. The unofficial short name for IEC TC 113 is Nano-electrotechnologies. The IEC TC 113 is interested in measurements, terminology, characterization, performance, reliability, durability, environment, health, and safety for nano-electrotechnologies.

The members of IEC TC 113 developed a list of applications for nano-electotechnologies shown below. Realizing that such a long list was not suitable for a survey, members of the IEC TC 113 Survey Project Team further refined the list to minimize overlap and created two lists—one for products and one for cross-cutting technologies. Each list has 8 items and is statistically more suitable for ranking by Survey respondents. Sub-section 2.1 Survey Structure and Methodology contains the products and cross-cutting technologies lists as Category Type 2 and Category Type 3, respectively.

**Applications of Nano-Electrotechnologies [6]**

- Analytical equipment and techniques for measurement of electrotechnical properties
- Fabrication tools for integrated circuits (electronic, photonics, and optoelectronic)
- Nano-structured sensors
- Nano-electronics, materials and devices
- Optoelectronics
- Optical materials and devices
- Organic (opto) electronics
- Magnetic materials and devices
- Radio frequency devices, components, and systems
- Electrodes with nano-structured surfaces
- Electrotechnical properties of nanotubes/nanowires
- Fuel cells
- Energy storage devices (e.g., batteries)
- Bioelectronic applications
- Nano-enabled solar cells

The scope of the IEC TC 113 concerns international standardization of those technologies relevant to electrical and electronic products and systems in the field of nanotechnology in close cooperation with other international groups working on standards and measurements for nano-electrotechnologies. These include, for example, other IEC committees, the International Standards Organization (ISO), the Institute of Electrical and Electronics Engineers, Semiconductor Equipment and Materials International (SEMI), and the International Technology Roadmap for Semiconductors Working Groups.

The focus of IEC TC 113 is on those products which use nano-electrotechnologies in one or more of their subassemblies or during the fabrication process. The IEC TC 113 will produce standards, technical specifications and technical reports to guide manufacturers and customers in situations where it is necessary to use an emerging technology under absence of complete knowledge to gain maximum confidence in the life cycle performance, reliability and operational safety of products. By so doing, the IEC TC 113 seeks to accelerate innovations and commercialization of nano-electrotechnologies.

## 2. Survey Origin and Development

Due to the large number of potential applications for nano-electrotechnologies and to the TC 113’s limited resources, there is a need to rank order future standardization work and make certain that the most important standards are developed first. To this end, the TC 113 Chairman’s Advisory Group (CAG) formed an international TC 113 Survey Project Team. The objective was to develop a Survey that would assist in identifying those nano-electrotechnology areas relevant to electronics and electrical products for which standards are critically needed to accelerate innovation.

The goal of the Survey was to begin building consensus among members of the international nano-electrotechnologies community on a framework leading to standards development. The expectation was that responses to the Survey would help prioritize TC 113’s actions over the next few years. Specific objectives of the survey were dictated by the governing principles shown in Table 1. Specifically, TC 113 would like to be able to 1) set procedures for ranking proposals and associated documents for new work in priority order; 2) identify members for work groups on standards and associated documents; and 3) make informed responses to proposals from IEC National Committees.

This Survey was the first step in developing the IEC TC 113 Nanoelectronics Standards Roadmap (INSR). Members of TC 113 will use the Survey results reported here as one of the inputs to the INSR that will establish a vision of market needs in terms of products, available technologies for nano-electrotechnologies and standards supporting invention, fabrication and use of products over their entire life cycle. The INSR will be an IEC integrated roadmap involving the stakeholders in the IEC. These stakeholders include the IEC National Committees that represent the electro-technical industries in their respective countries as well as IEC TC 113 liaison organizations like the Institute for Electrical and Electronics Engineers (IEEE) and SEMI. The INSR will be developed by a newly formed Task Group in IEC TC 113 and be published as a Technical Report. The INSR will be revised biannually. The officers of IEC intend that the INSR will complement other publicly available roadmaps such as the International Technology Roadmap for Semiconductors (ITRS) and the IEEE Nanoelectronics Standards Roadmap.

### 2.1 Survey Structure and Methodology

The authors collaborated with members of the IEC TC 113 Chairman’s Advisory Group (CAG) to prepare the text for a web-based Survey. The Survey was designed to determine priority rankings of the needs for standards and their supporting measurements that should be considered by IEC TC 113. Appendix A contains the full text for the Survey.

Once we completed the text and formats for the outputs from the Survey, the text was converted into HTML format for Internet access. SelectSurvey.NET version 2.8.7 was used as the platform for the on-line Survey, which was on-line from May 10, 2008 to December 15, 2008 at http://www.energetics.com/IEC-NISTSurvey/index.html.

The Survey opened with demographic questions that had drop down lists for selecting responses:

1. How would you describe the nature of your work in nano-electrotechnologies?
2. What is the type of institution where you are primarily employed?
3. Please select your country of primary employment.

Note that the country drop-down list contained countries that are members of IEC TC 113. If a respondent’s country was not on that list, they were invited to write in a country. Section 3 discusses the Survey demographics in more detail, including the countries of primary employment.

Survey respondents were then asked to rank in priority order the items listed in each of five category types from 1 to *n_i_*, where *n_i_* is the number of items in the category type *i* under consideration and *i* = 1, 2, …, or 5. The rank of 1 denotes the highest priority or most significant and the rank of *n_i_* denotes the lowest priority or least significant. The Survey software, SelectSurvey.NET 2.8.7, presented each respondent the items for a given category type in random order. This helped to avoid potential biases in the data that might arise if each respondent saw the items to be ranked in the same order. The five category types employed in the Survey and the relevant Governing Principle from Table 1 are as follows:

1. Nano-electrotechnology Properties (*Governing Principle II*) (*n*_1_ = 6)
  – *Electronic and Electrical* [Electronic]
  – *Optical* [Optical]
  – *Biological* [Biological]
  – *Chemical* [Chemical]
  – *Radio Frequency* [Radio]
  – *Magnetic* [Magnetic]
2. Nano-electrotechnology Taxonomy: Products (*Governing Principle I*) (*n*_2_ = 8)
  – *Energy* (*production, conversion, and storage*) [Energy]
  – *Medical Products* [Medical]
  – *Computers* (*PDA and similar, laptop, desktop, mainframe*) *and Computer Peripherals* (*print-ers, monitors/displays, etc.*) [Computers]
  – *Telecommunication and Data Communications* (*wireless and wired-physical connection*) [Telecom]
  – *Security and Emergency Response Devices and Applications* [Security]
  – *Multimedia Consumer Electronics* [Multimedia]
  – *Household and Consumer Applications* [Household]
  – *Transportation* (*sea/water, ground, air, space*) [Transportation]
3. Nano-electrotechnology Taxonomy: Cross-Cutting Technologies (*Governing Principle I*) (*n*_3_ = 8)
  – *Sensors* (*chemical, physical, mechanical, etc.*) [Sensors]
  – *Fabrication tools for integrated circuits* (*electronic, photonic, optoelectronic, and mechanical*) [Fab. Tools]
  – *Nano-electromechanical systems* [NEMS]
  – *Performance and reliability assessment for nanoelectronics* [Performance]
  – *Analytical equipment and techniques for measurements of electro-technical properties* [Analytic Eq.]
  – *Environment, Health, and Safety (EHS) applications and effects* [EHS]
  – *Instrumentation (test equipment and industrial process control for use in fabrication)* [Instrumentation]
  – *Optical technologies (optoelectronics and illumination)* [Optical Tech.]
4. IEC General Discipline Areas (*Governing Principle III*) (*n*_4_ = 6)
  – *Measurement and Performance* [Measurement]
  – *Design and Development* [Design]
  – *Health, Safety and Environment (HSE)* [HSE]
  – *Dependability and Reliability* [Dependability]
  – *Electromagnetic Compatibility* [Compatibility]
  – *Terminology, Nomenclature, and Symbols* [Terminology]
5. Stages of Economic Model (*Governing Principle IV*) (*n*_5_ = 6)
  – *Basic Technical Research* [Research]
  – *Technology Development* (*prototype development)* [Development]
  – *Initial deployment* [Deployment]
  – *Commercialization* (*large-scale, high-volume manufacturing*) [Commercialization]
  – *End of initial use by the Customers-Consumers* (*End of Initial Usefullness*) [End-of-Usefullness]
  – *End-of-Life* (*disposing and recycling*) [End-of-Life]

The square bracket after each of the above items contains the abbreviation for that item used in the figures that follow in Secs. 4 to 6.

The international community tends to use different orderings of the words environment, health, and safety, and hence, different orderings of the letters E, H, and S in related acronyms. To distinguish in this paper between the Cross-Cutting Technology and the Discipline Area, we use the acronym *EHS* for the Cross-Cutting Technology of *Environment, Health, and Safety Applications and Effects* and the acronym *HSE* for the IEC General Discipline Area of *Health, Safety, and Environment.*

After asking respondents to rank the above items in priority order, the survey asked them to express their interest in volunteering to help the IEC TC 113 and to submit general comments concerning the Survey.

### 2.2 Survey Advertisements

Table 2 lists the many organizations that contributed to promoting the Survey. The officers, editors, and staff of the organizations listed therein distributed emails to their respective members and/or wrote articles about the Survey that invited their members and readers to complete the on-line Survey. In addition, the Survey was advertised at several conferences where those attending would be associated in some way with nano-electrotechnologies

These efforts attracted more than 600 respondents to the Survey. Section 3, Survey Demographics, provides a complete breakdown of those actually completing the Survey in its entirety. In addition, the Survey was open for an extended period (7 months) and re-advertised to gain a larger sample size, encourage a greater number of participants from more countries, and help enhance the statistical credibility of the responses and results. The number of completed responses increased from 205 in August 2008 to 459 in December 2008—a 223 % increase.

## 3. Survey Demographics

**Fig. 1:**
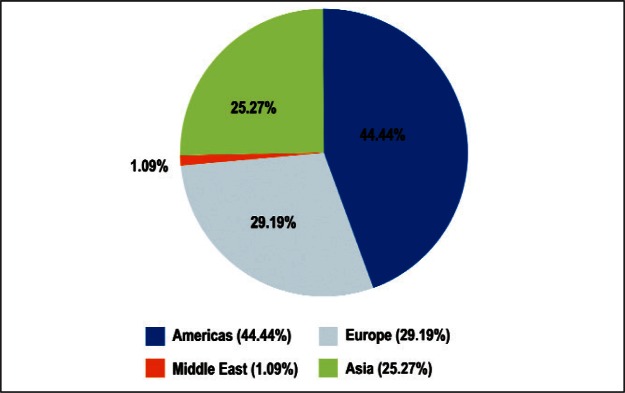
Demographics of Survey Respondents.

In total, 459 respondents from 45 countries, listed in Table 3, volunteered to complete the Survey in its entirety. Here a complete response is defined as a response for which all three of the demographic questions and all five of the ranking categories were completed. We restrict our analyses to these completed responses. As shown in Fig. 1, 44.4 % came from the Americas, 29.2 % from Europe, 25.3 % from Asia, and 1.1 % from the Middle East.

The respondents self-reported as practicing in countries representing most large geographic areas. We do not attempt to draw inferences about any of the demographic sub-categories as such. For example, we do not attempt to weigh demographic sub-categories by response rate to achieve a consistent weighting in the consensus average. Rather, survey respondents are a self-selected group with interests and opinions for improving standards and measurements that support innovations and commercialization of nano-electrotechnologies. Their demographic data is used primarily for categorical purposes.

**Fig. 2:**
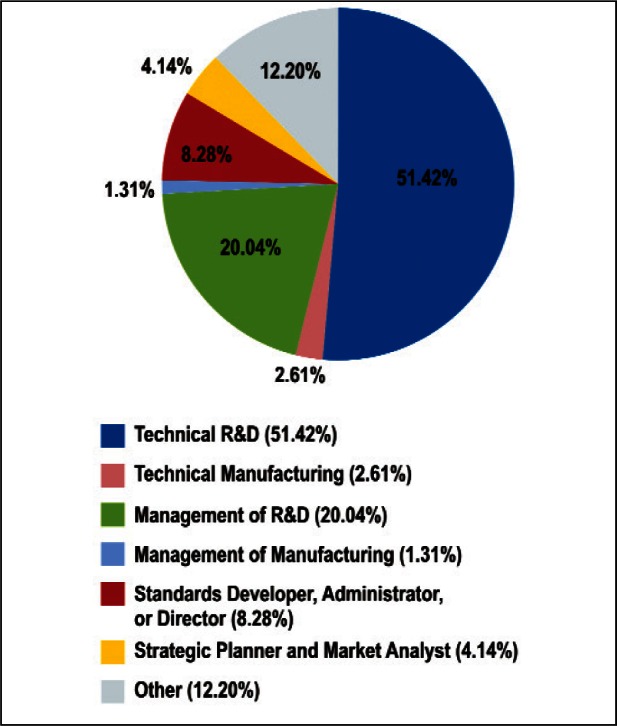
Distribution of Survey Respondents: Nature of Work.

**Fig. 3:**
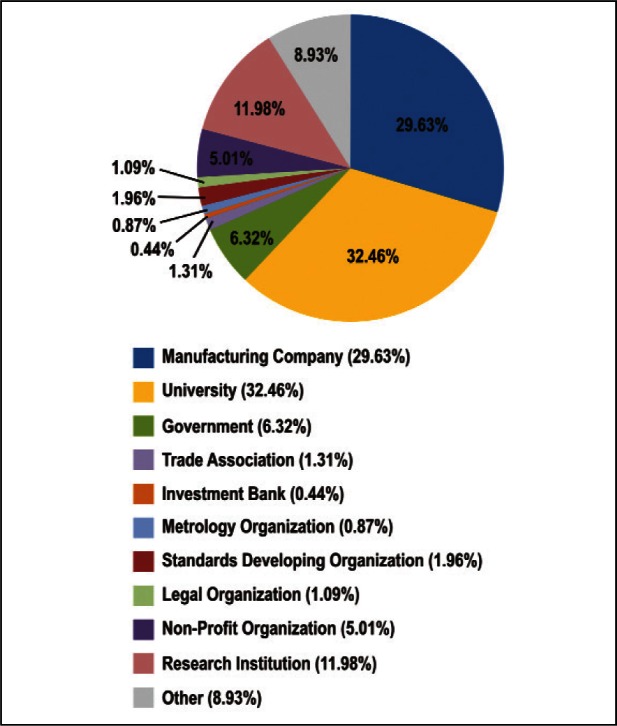
Distribution of Survey Respondents: Place of Employment.

As shown in Figs. 2 and 3, the Survey respondents represented a broad cross-section of the nano-electrotechnologies community. The nature of work represented spans technical R&D and management, manufacturing, standards development, strategic planning, and market analyses. Places of employment of respondents included manufacturing companies, universities, governments, trade associations, banks, standards and metrology organizations, and legal organizations.

The largest categories represented in the nature of work were both research-related: Technical R&D and Management of R&D. This is largely indicative of the emerging nature of nanotechnology and the significant amount of research and development ongoing in this field. While new products are emerging regularly, many others are still in the early development and proof-of-concept phases.

The largest percent of respondents were from universities, followed by those from manufacturing companies and a significant number from research institutions. This reflects a strong research and development focus in the field of nanotechnology, as well as significant interest in new product development and manufacture.

The small percentage, about 3 %, of respondents from metrology organizations and standards development organizations could indicate that the majority of responders were users of measurement technology, either for research or product development. A more significant portion of respondents, about 14 %, came from government and non-profits.

## 4. Priorities Analysis

One of the primary goals of the survey was to determine a consensus prioritization among the items listed for each of the category types. With this goal in mind, the Survey required the respondents to rank all items for each of the five category types, with no ties allowed. Tallying the results from all respondents provides a priority rank distribution in a given category type. In this analysis, we consider the distributions based on all respondents, but do not consider various demographic sub-categories.

Considering the sample size and the statistical nature of the distributions of responses, especially since some distributions were strongly bimodal, we do not give the precise rank importance of each and every item included in the Survey. Instead, we introduce a coarser analysis in which we place subsets of the Survey items into sub-groups and then rank the sub-groups in priority order. This coarser analysis is an alternative procedure described in more detail in the recent *Analysis of ISCD-NIST Survey for Bone Health* [7]. We find that this sub-grouping of Survey items offers a prioritization scheme that is reasonably consistent across several Survey categories.

### 4.1 Ordinal Statistics and Concordance

**Fig. 4:**
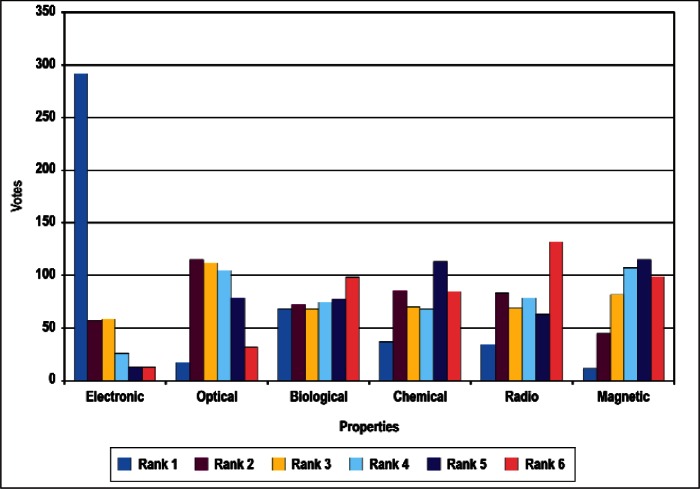
Rank Distribution for Properties Category.

In this section, we present preliminary statistical analyses. As noted above, we restrict the discussions to results treating all respondents as a single group. Figures 4 through 8 provide histograms of the vote (ballot) distributions from all Survey respondents for each of the five category types. In each figure, each of the *n_i_* items to be ranked in that category type has *n_i_* bars associated with it. The first bar on the left is the number of respondents who gave that item a rank of 1. The next bar is the number of respondents who gave that item a rank of 2, and so forth. A rank of 1 indicates the highest priority and a rank of *n_i_* is the lowest priority.

Figures 9 through 13 give the medians, first quartiles, third quartiles, and 95 % confidence intervals (CI) for each of the priority ranked items in the five category types. Appendix B contains the formula given by Eq. (B.1) that we use for computing the 95 % CI values, i.e., the uncertainty in the median estimate. The use of median as a measure of central tendency, as opposed to mean, is more appropriate for the ordinal nature of the rank data [8].

In each of these 5 figures, we give the *n_i_* category type *i* items in sorted order, with the left most item considered to be the most important. The thick-horizontal lines in the vertically-oriented shaded boxes indicate the median values. The vertical extents of the larger shaded boxes correspond to the first and third quartiles. The vertical extents of the smaller boxes inside the larger shaded boxes indicate the 95 % confidence intervals for the uncertainty estimate of the median as computed by Eq. (B.1) in Appendix B.

We computed Friedman’s statistic to assess the degree of distinction between items. Our analysis follows Lehmann [9] and details are provided in Appendix B. Friedman’s statistic is designed to test the null hypothesis, namely,

*H*_0_ = “Voters-respondents randomly assigned ranks to the items with equal probability.”

In other words, when *H*_0_ is true, then the distribution of votes reflects no discernible preference among items. To test *H*_0_, we compute Friedman’s statistic *Q* according to Eq. (B.2) in Appendix B and compare the value against the null distribution by way of the confidence *p*-value. One interpretation of the *p*-value in relation to an observed value, *Q_obs_*, is that if *H*_0_ were true, then one would expect a value of *Q* greater than or equal to *Q_obs_* with probability *p*.

We use Eq. B.4 in Appendix B to compute the *p*-value. We find that for all respondents we can reject *H*_0_ with more than 99 % confidence (*p* < 0.01). Such a conclusion is consistent with the observation that the estimates of the median ranks for all of the items, e.g., Fig. 11, are such that the 95 % confidence intervals (B.1) for all *n_i_* items do not overlap. This lack of overlap provides evidence that there are perceived differences among the *n_i_* items. The exceptions to this are likely to be when the conditions given in Appendix B are not met.

In summary, although the histogram plots such as that shown in Figs. 4 through 8 do not reveal obvious structure, the distributions of ranks suggest that it is unlikely that they were assigned randomly with equal preference to all items. We discuss our strategy for determining global consensus ranks in the next subsection.

Tables 4 through 8 show the consensus priorities for each of the five category types as determined by a traditionally weighted scoring technique called the Borda count [10]. Applying this procedure to the present Survey category types we assign the following score-weights: the first-placed items (highest priority or most significant) on every ballot receive scores of *n_i_*, the second-placed items receive scores of *n_i_* − 1, and so forth, until the lowest priority or least significant items on the ballot receive scores of 1. We assign the scores to each ballot individually, and then sum over all ballots within the category type of interest. We rank the items in descending order by the Borda score, i.e., the highest score is the “winner.” In short, the Borda score is a weighted mean with a particular assignment of weights to ballot positions. We refer throughout this paper to these Borda count orderings as the “global consensus” orderings.

The global consensus order may not be the same as the order when only rank 1 votes are considered. For example, Fabrication Tools in Table 6 received 109 rank 1 votes, 61 rank 2 votes,…, and 44 rank 8 votes. All of the remaining 7 items in Table 6 received fewer than 109 rank 1 votes. We estimate the median rank of the underlying random variable to be 3 ± 0.29. The global consensus is that Fabrication Tools is second to Sensor as a priority activity for IEC TC 113 to promote nano-electrotechnologies.

### 4.2 Rank Prioritizations

Aggregating a collection of rankings to determine a consensus rank is a well-known problem in voting and social choice theory [10,11]. There are several competing algorithms and there is no clear “optimal strategy” among them. As discussed in the previous paragraphs, we select a traditional positional weighting scheme referred to as a Borda method. We emphasize that both the choice of a positional scoring method, and subsequently the selection of weights to be applied, can affect the results. For example returning to Table 6, whereas the Fabrication Tools receives the most rank 1 votes, the Borda scoring scheme values the relatively large number of second and third place votes received by Sensors to the extent that the latter edges out the former. One could envision alternative weighting schemes that allocates higher value to first-placed ranks relative to the middle-placed ranks than does the arithmetic sequence *n_i_*, *n_i_* − 1,, …, 1. For example, in such cases the consensus prioritization between Fabrication Tools and Sensors could transpose.

The final prioritizations in their every detail are not very precise. However, slightly coarser analyses suggest themselves as being possible and agreeable to all respondents. In this re-factoring or re-grouping of the *n_i_* items in each category type *i*, we rank sub-groups of items for each category type by their respective median values and then order the items within a sub-group by their respective Borda global consensus count order. We list the highest priority category type sub-group first in the following prioritizations:

1. Properties (Figure 9 and Table 4)
  Sub-Group 1 - *Electronic and Electrical*
  Sub-Group 2 - *Optical*
  Sub-Group 3 - *Biological; Chemical; Radio Frequency; and Magnetic*
2. Products (Figure 10 and Table 5)
  Sub-Group 1 - *Energy; Medical Products; and Computers*
  Sub-Group 2 – *Telecommunications*
  Sub-Group 3 - *Security and Emergency Response and Multimedia Consumer Electronics*
  Sub-Group 4 - *Household and Consumer Applications*
  Sub-Group 5 - *Transportation*
3. Cross-Cutting Technologies (Figure 11 and Table 6)
  Sub-Group 1 - *Sensors and Fabrication Tools*
  Sub-Group 2 - *Nano-electromechanical Systems*
  Sub-Group 3 - *Performance Assessment*; *Analytical Equipment; EHS; Instrumentation; and Optical Technologies*
4. Discipline Areas (Figure 12 and Table 7)
  Sub-Group 1 - *Measurement and Performance*
  Sub-Group 2 - *Design and Development; HSE; and Dependability and Reliability*
  Sub-Group 3 - *Electromagnetic Compatibility and Terminology and Symbols*
5. Stages of the Linear Economic Model (Figure 13 and Table 8)
  Sub-Group 1 - *Basic Technical Research and Technology Development*
  Sub-Group 2 - *Initial Deployment and Commercialization*
  Sub-Group 3 - *End-use by the Customer-Consumer and End-of-Life*

The above five prioritizations suggest that IEC TC 113 should focus in the short-term on standards and measurements for electronic and electrical properties of sensors and fabrication tools that support performance assessments and measurements of nano-technology sub-assemblies used in energy, medical, and computer products.

## 5. Correlations Analysis

Any correlation analyses among the several items in the five category types (Properties, Products, Cross-Cutting Technologies, Discipline Areas, and Stages of the Linear Economic Model) and in the three demographic items (Country-region, Nature of Work, and Employment Institution) should meet the validity conditions given in Appendix B. Specifically, the validity conditions include: 1) a large enough sample size, *N_sample_*, 2) a small enough Kendall’s W, and 3) a vanishingly small confidence *p*-value. Our approach for deciding which correlations are likely to satisfy the above validity conditions begins by correlating those items that have a large enough number of ranked 1 votes within a category type with all of the items in another category type. For example, among the eight items in the category type Products, *Energy* received the most rank 1 votes, namely 130. *Computers and Medical Products* with rank 1 votes of 109 and 85, respectively, followed Energy. Figure 14 then shows how the 130 *Energy* respondents ranked the 8 items in the category type Cross-Cutting Technologies.

Figures 14 through 27 show the correlation results for the following comparisons:

- Products: *Energy, Computers, Medical and Telecommunication and Data Communications* versus Cross-Cutting Technologies (Figs. 14 to 17)
- General Discipline Area: *Design and Development, Health, Safety, and Environment (HSE)*, and *Measurement and Performance* versus Products (Figs. 18 to 20)
- General Discipline Area: *Design and Development, Health, Safety, and Environment (HSE)*, and *Measurement and Performance* versus Cross-Cutting Technologies (Figs. 21 to 23)
- Nature of Work: *Technical R&D and Management R&D* versus Stages of the Economic Model (Figs. 24 and 25)
- Employment Institution: Universities and Manufacturing Companies versus Cross-Cutting Technologies (Figs. 26 and 27).

The confidence *p*-values failed to approach zero for two of the correlations that we considered: 1) Nature of Work: Standards Developer, Administrator, or Director of R&D versus Stages of the Economic Model and 2) Employment Institution: Research Institutions versus Cross-Cutting Technologies. The *p*-values for these two correlations indicate that the sample sizes may not be large enough for acceptable statistical analyses. The distributions of rankings in this Survey suggest that the validity conditions may not be met in correlations with samples sizes less than about 85.

**Fig. 6:**
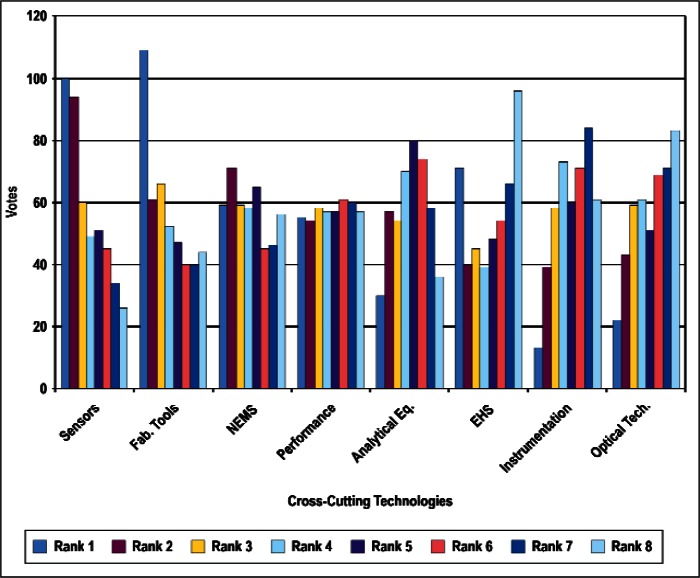
Rank Distribution of Cross-Cutting Technologies.

Comparing the correlation rankings given in Figs. 14 to 27 reveals many transpositions of priority rankings. An interesting result is that the bimodal distribution of item Cross-Cutting Technologies: *Environment, Health, and Safety (EHS) Applications and Effects* that appears in Fig. 6 and Table 6 is further supported by correlations. In statistics, a bimodal distribution is a probability distribution with two different modes (e.g., peaks or values) that occur more frequently than neighboring values. As shown in Fig. 15, Products: *Computers* versus Cross-Cutting Technologies, the item *EHS Applications and Effects* ranks last in priority. Whereas in the correlation shown in Fig. 16, Products: *Medical Products* versus Cross-Cutting Technologies, the item *EHS Applications and Effects* ranks first in priority.

The bimodal distribution of the Cross-Cutting Technologies item *EHS Applications and Effects* demonstrates what we might expect: from a medical products viewpoint, *Environment, Health and Safety* are of paramount importance; from the viewpoint of a manufacturer of computers, the issues that directly affect production (fabrication of circuits, sensors, performance, and reliability) are of most importance. Table 9 illustrates the statistical results that support evidence of the bimodal distribution. Additionally, while the IEC Discipline Area item of *Health, Safety and Environment* in general appears to be important across many groups, it is less important than some of the disciplines relevant to earlier stages of the product cycle (e.g., *Design and Development*) and production stages (*Measurement and Performance*).

Figures 24 and 25 illustrate the correlation of the largest number of responders in terms of nature of work (Technical R&D and Management R&D) versus the Stages of the Economic Model. Both groups of respondents indicated that *Basic Technical Research and Technology Development* were among their top ranked Stage of the Economic Model, with less emphasis placed on the stages related to technology C*ommercialization and Initial Deployment*. This is indicative of the nature of the respondent demographic—over 70 % of respondents were listed as being in Technical R&D or Management of R&D positions (Fig. 2).

In Figs. 26 and 27, the correlation between the largest number of responders in employment institutions (Universities and Manufacturing Companies) versus Cross-Cutting Technologies illustrates both institution types have a keen interest in *Sensors, Fabrication Tools for Integrated Circuits*, and *Nano-electromechanical Systems.* In general across all the correlations, *Sensors* and *Fabrication Tools for Integrated Circuits* were ranked among the first three choices, regardless of category. The correlations as a result support the overall conclusion that the IEC TC 113 should focus initially on standards and measurements for electronic and electrical properties of sensors and fabrication tools.

## 6. Conclusions

Our analyses suggest that the majority of the 459 respondents agree with the following statements:

1. The most important items on which IEC TC 113 should work are those items included in the Sub-Groups 1 for each of the category types listed in the Ranked Prioritizations Sub-Section; namely, *Electronic and Electrical* properties of *Sensors* and *Fabrication Tools* used to manufacture *Medical*, *Computer*, and *Energy* products.
2. Because the time frame of the Survey was the short-term, the critical discipline areas for IEC TC 113 technical experts will be initially *Measurements and Performance* assessments that include metrics for determining reliability and durability of nano-electrotechnology enabled products and systems.
3. IEC TC 113 members should focus their work initially on those standards and measurements that contribute to advances in the economic stages of *Technical Research* and *Technology Development* related to the fabrication of nano-electrotechnology enabled products and systems.

The Survey respondents as a whole do not agree on the relative importance of the Cross-Cutting Technology item *EHS Applications and Effects*. Almost as many respondents said that *EHS Applications and Effects* were most important as said that they were least important from among the eight items listed for Cross-Cutting Technologies. Furthermore, those respondents who said *Medical* products were most important also said *EHS Applications and Effects* were most important. Whereas, those who said *Energy, Computer*, and *Telecommunication and Data Communications* products were most important said *EHS Applications and Effects* were least important. This apparent dependence of the relative importance of *EHS Applications and Effects* on specific products requires consideration in the INSR and may warrant additional investigations.

The data samples for correlations of Cross-Cutting Technology: EHS Applications and Effects with the remaining four Product items Security and Emergency Response Devices, Multimedia Consumer Electronics, Household and Consumer Applications, and Transportation are such that the respective 95 % Confidence Intervals are too large and thereby do not allow us to reach statistically defensible statements. Combining the major results from Figs. 14 to 17, we use the schematic in Fig. 28 to show graphically the above dependence for the four Product items that have acceptable 95 % Confidence Intervals. Namely, the 71 respondents who gave EHS Applications and Effects the highest priority assigned highest priority to Medical Products. We order for the figures that follow, beginning with Fig. 28, the ranked items in ascending Borda rank. The item at the top has the highest priority rank and the item at the bottom has the lowest priority rank.

Figures 29 and 30 summarize respectively the correlations of the Discipline Area item *Health, Safety and Environment* and the Cross-Cutting Technology item *EHS Application and Effects* with the stages of the Economic Model. The 129 respondents who ranked the Discipline Area item *Health, Safety and Environment* the highest and the 71 respondents who ranked the Cross-Cutting Technology item *EHS Application and Effect* the highest assigned the highest priorities to the Economic Model stages of *Basic Technical Research* and *Techno-logy Development* and the lowest priority to *Commercialization.*

Finally, Figs. 31 and 32 summarize respectively the correlation of the Products item *Medical Products* with Cross-Cutting Technologies and the correlation of the Cross-Cutting Technologies item *Sensors* with Products. The 85 respondents who ranked the Products item *Medical Products* the highest assigned the highest priorities to the Cross-Cutting Technologies *EHS Applications and Effects* and *Sensors*. The 100 respondents who ranked the Cross-Cutting Technology item *Sensors* the highest assigned the highest priorities to *Energy* and *Medical Products*. Combining these two sets of correlation figures for *Medical Products* and *Sensors* suggests a consensus among many respondents that standards and measurements for bio-sensors enabled by nano-electrotechnologies have very high priorities.

We intended that this broadly-based Survey elicit the views of the nano-electrotechnologies community as to ways for advancing innovations and commercialization. The goals of this survey were to determine the extent of consensus from the nano-electrotechnologies community around the four governing principles listed previously. From the survey, we surmise that the IEC TC 113 should focus initially on R&D standards and measurements for electronic and electrical properties of sensors and fabrication tools that support performance assessments of nano-technology enabled sub-assemblies used in energy, medical, and computer products.

**Fig. 9:**
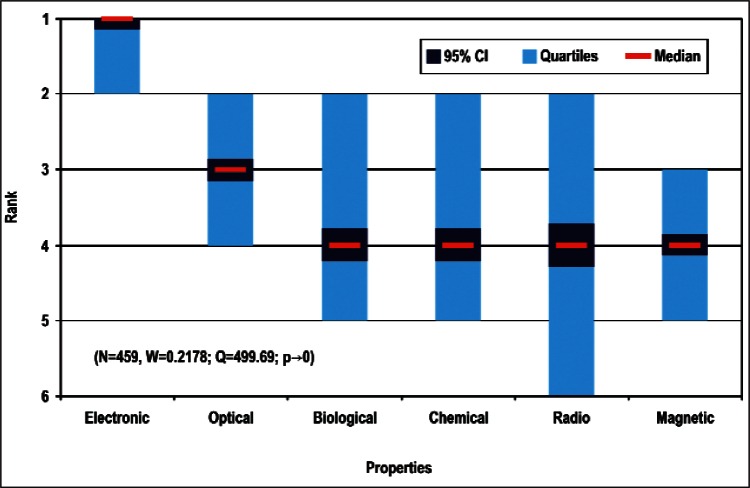
Medians and Confidence Intervals for Property Rankings.

**Fig. 10:**
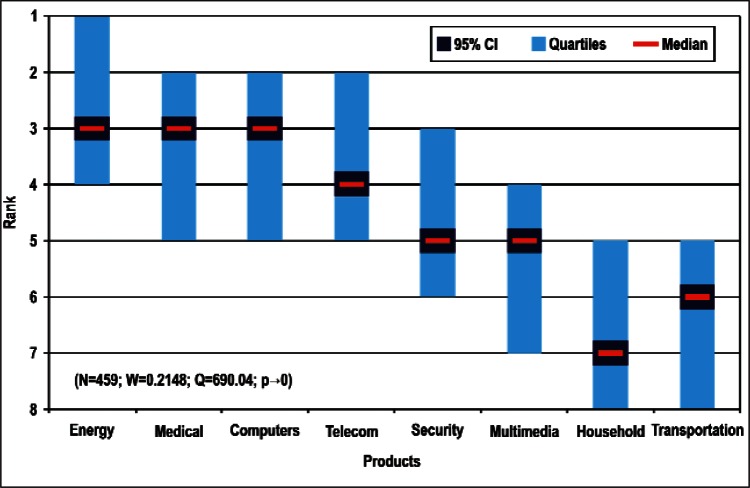
Medians and Confidence Intervals for Product Rankings.

**Fig. 11:**
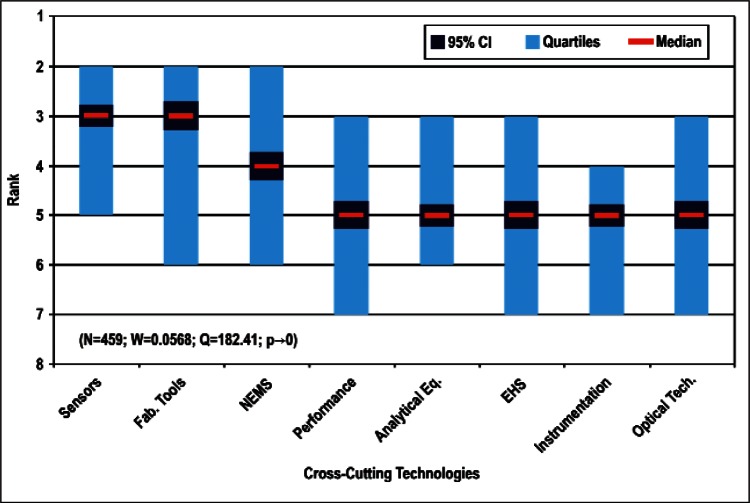
Medians and Confidence Intervals for Cross-Cutting Technology Rankings.

**Fig. 12:**
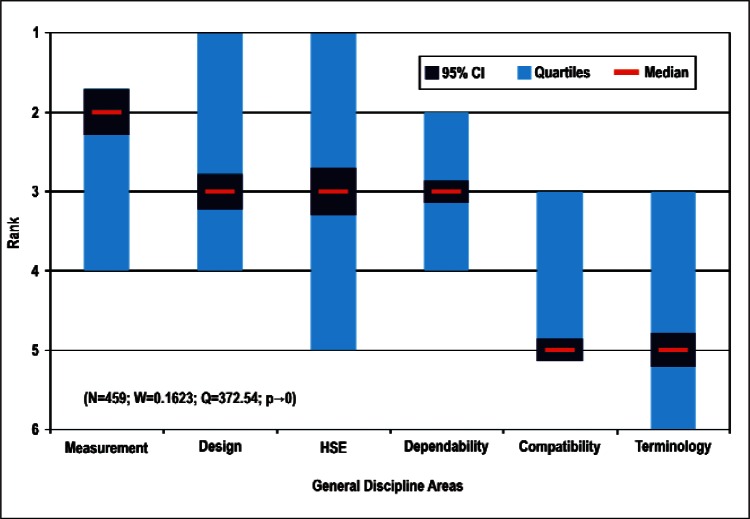
Medians and Confidence Intervals for General Discipline Rankings.

**Fig. 13:**
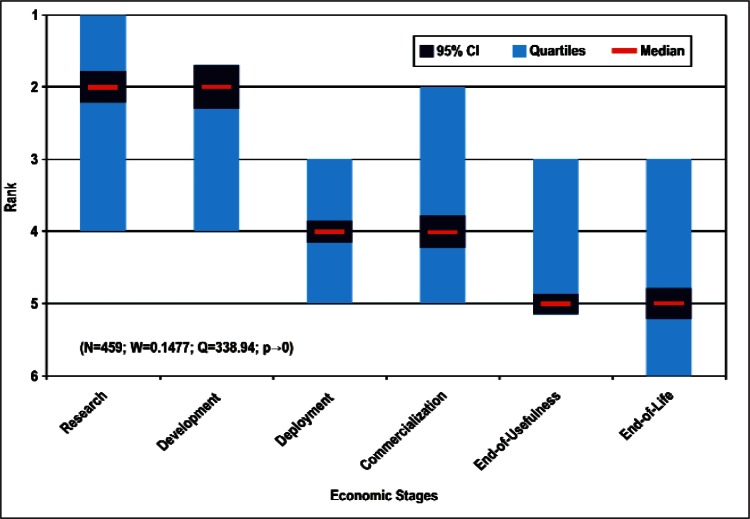
Medians and Confidence Intervals for Economic Stage Rankings.

**Fig. 14:**
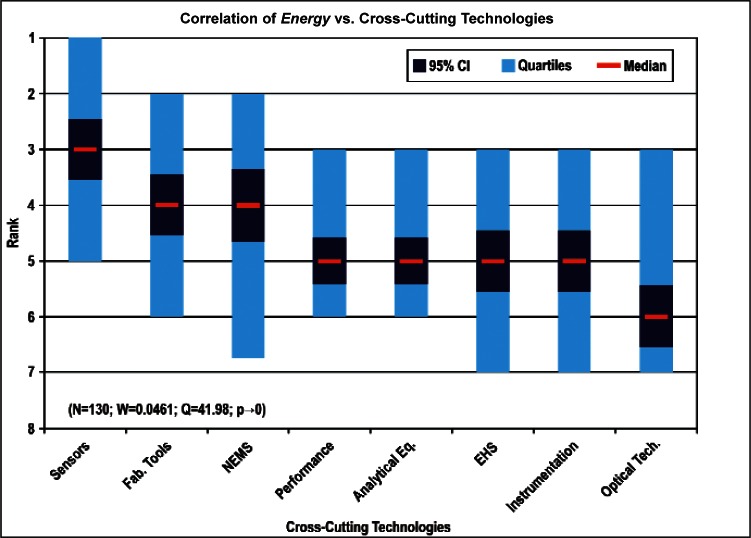


**Fig. 15:**
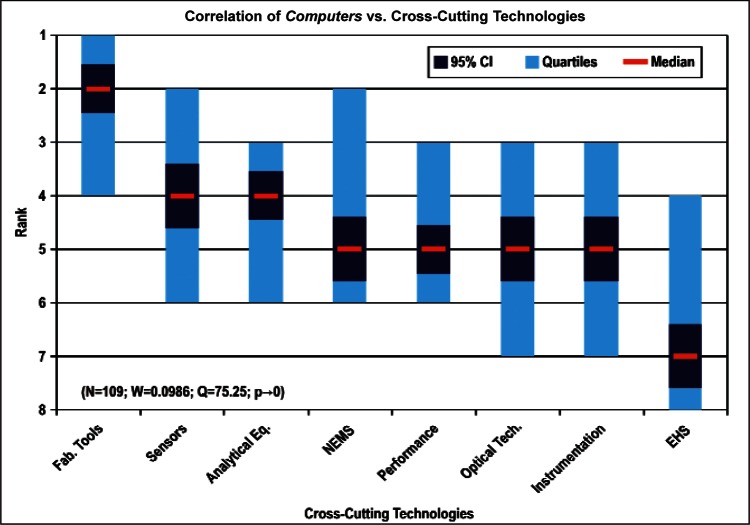


**Fig. 16:**
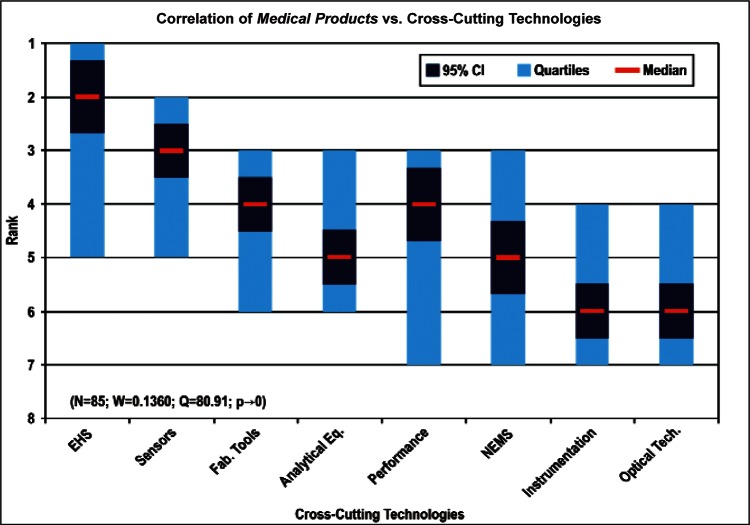


**Fig. 17:**
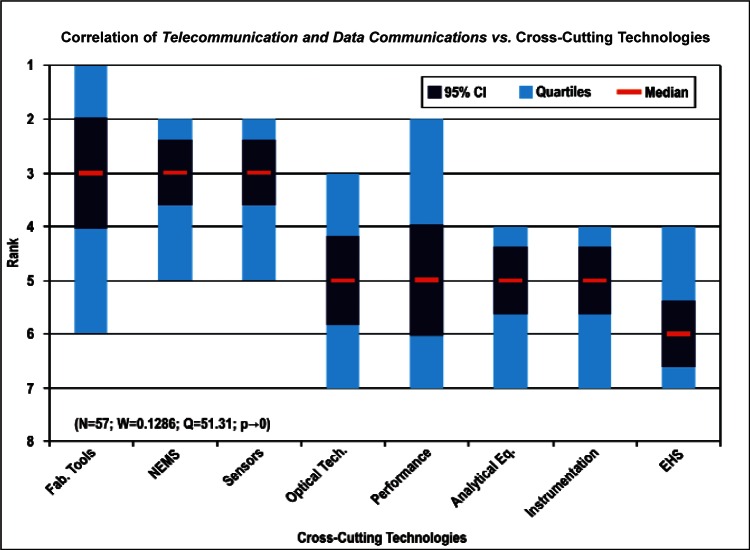


**Fig. 18:**
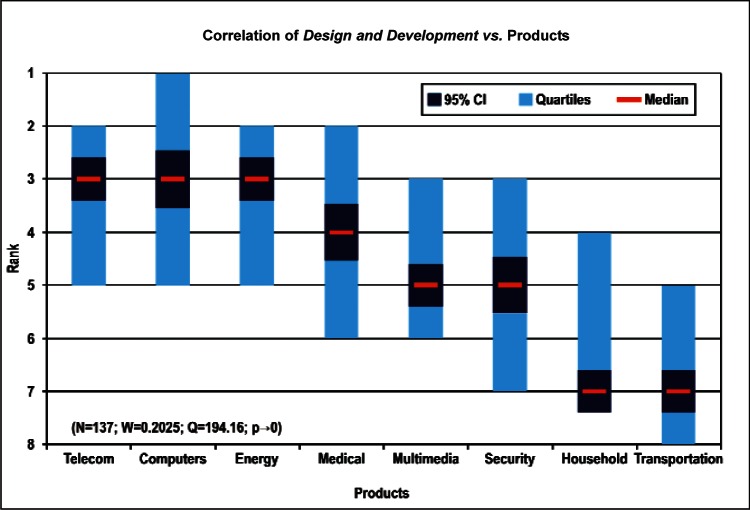


**Fig. 19:**
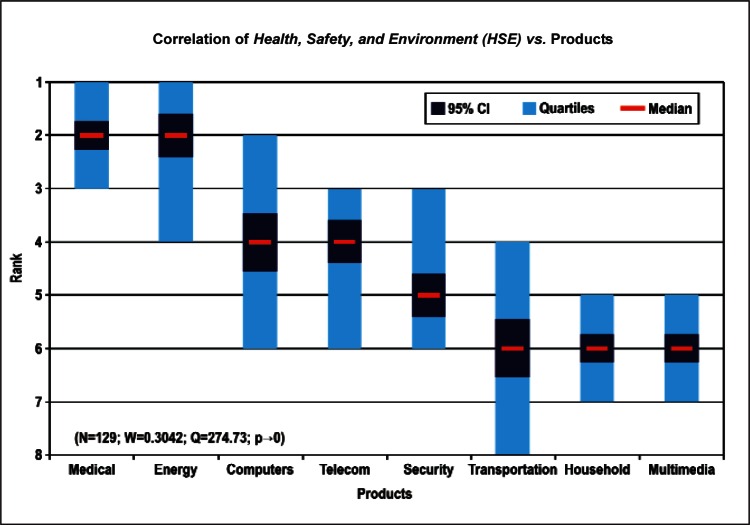


**Fig. 20:**
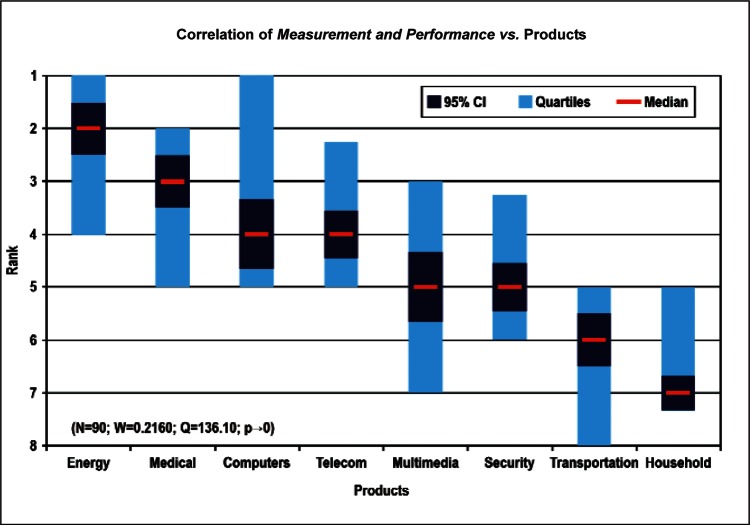


**Fig. 21:**
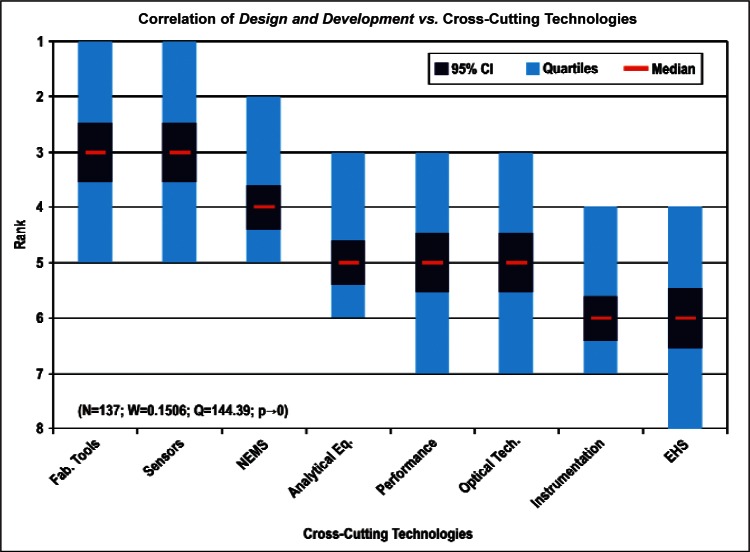


**Fig. 22:**
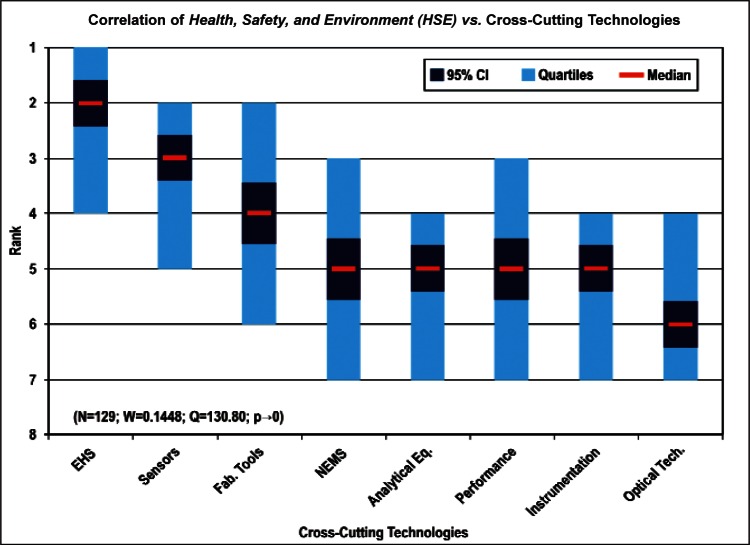


**Fig. 23:**
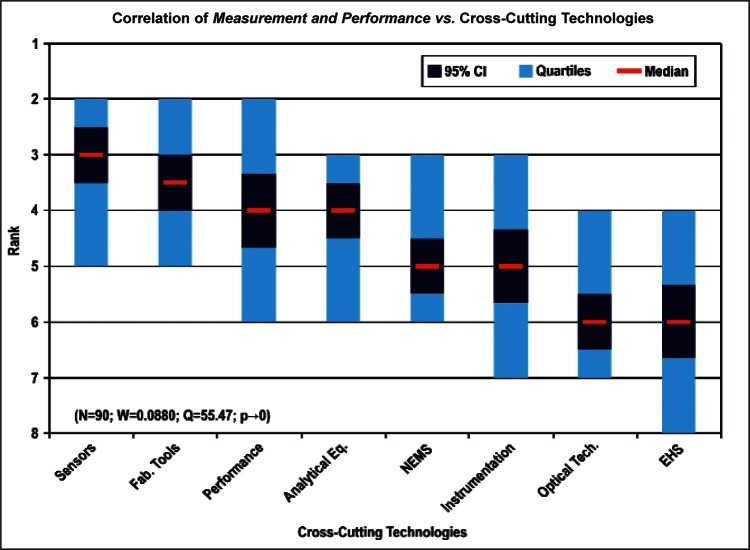


**Fig. 24:**
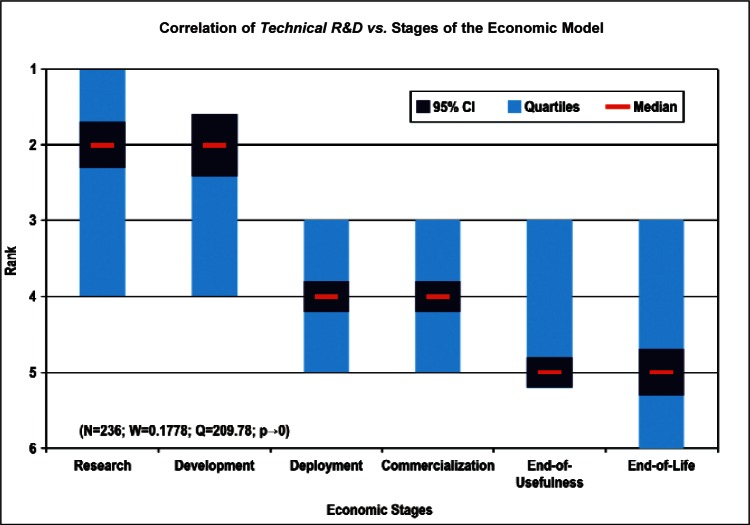


**Fig. 25:**
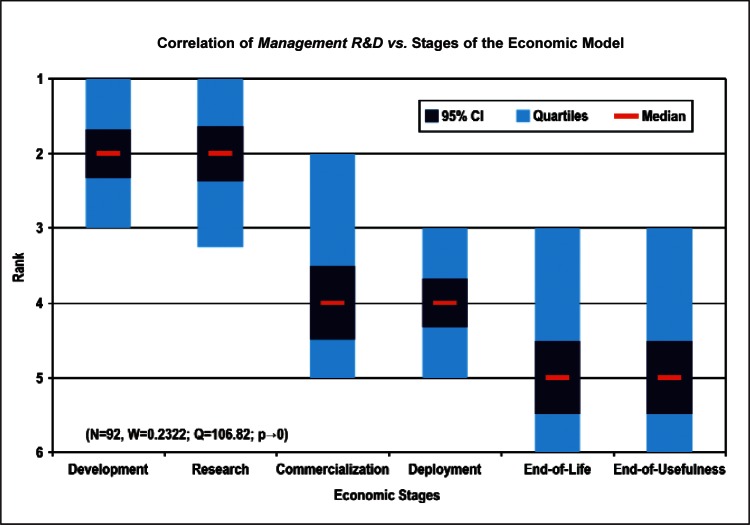


**Fig. 26:**
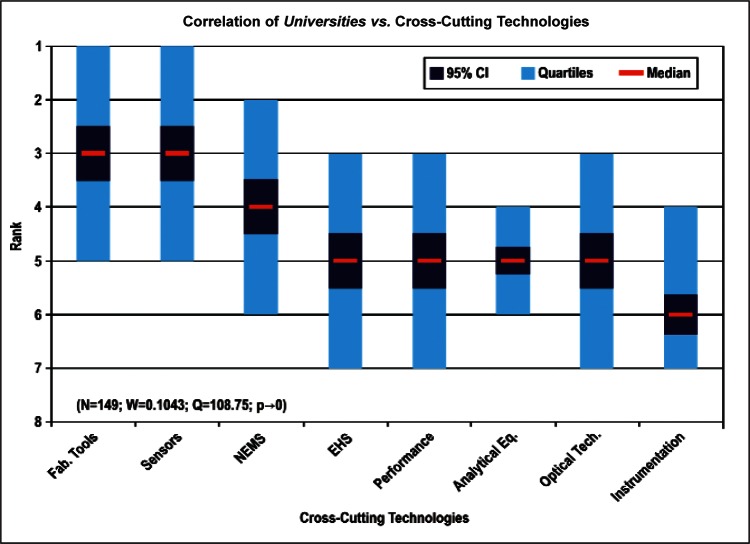


**Fig. 27:**
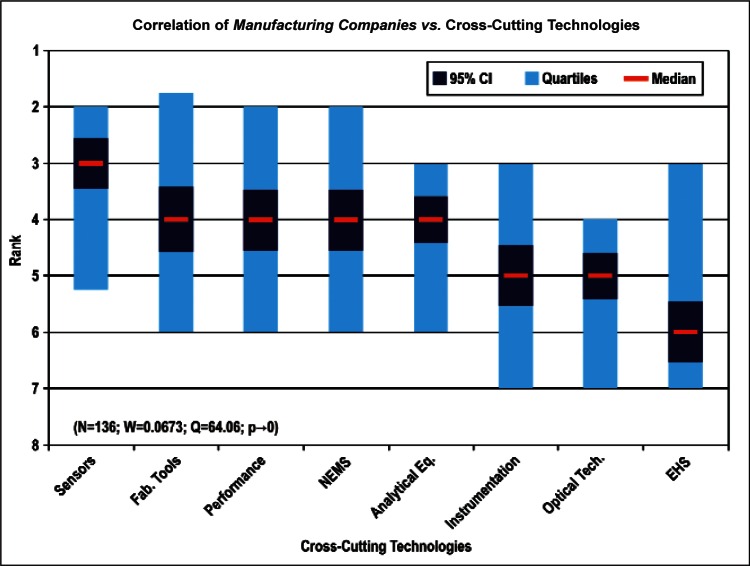


**Fig. 28:**
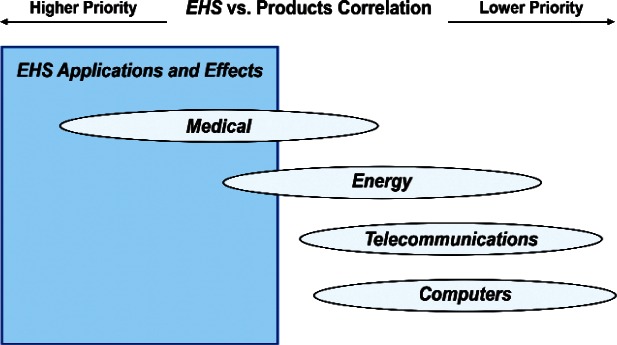
Schematic of the correlation of the relative importance of Cross-Cutting Technology item *EHS Applications and Effects* with four of the eight Product items.

**Fig. 29:**
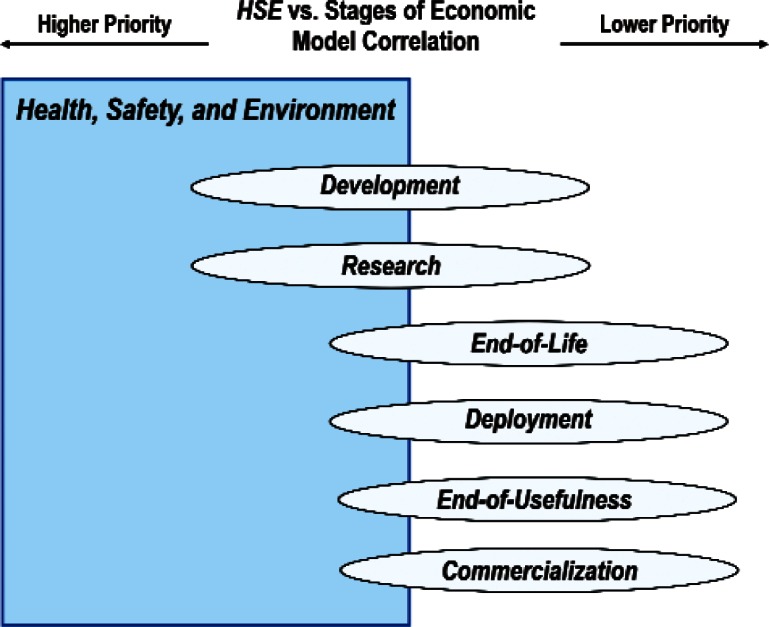
Schematic of the correlation of the relative importance of Discipline Area item *Health, Safety, and Environment* with the Economic Model stages.

**Fig. 30:**
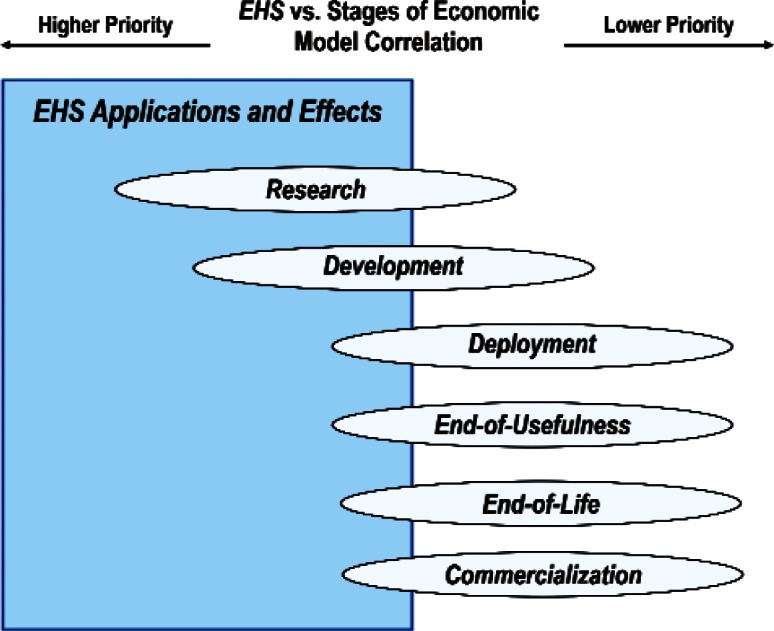
Schematic of the correlation of the relative importance of Cross-Cutting Technology item *EHS Applications and Effects* with the Economic Model stages.

**Fig. 31:**
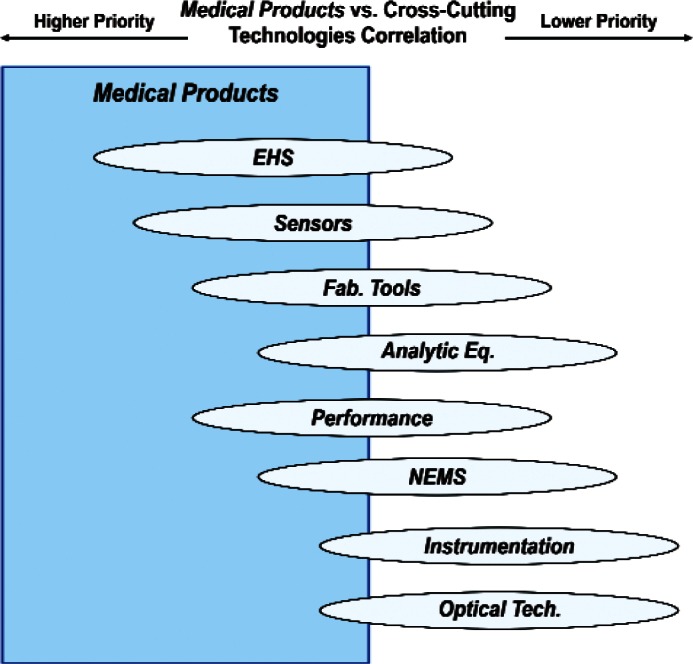
Schematic of the correlation of the relative importance of Products item *Medical Products* with the Cross-Cutting Technologies category.

**Fig. 32:**
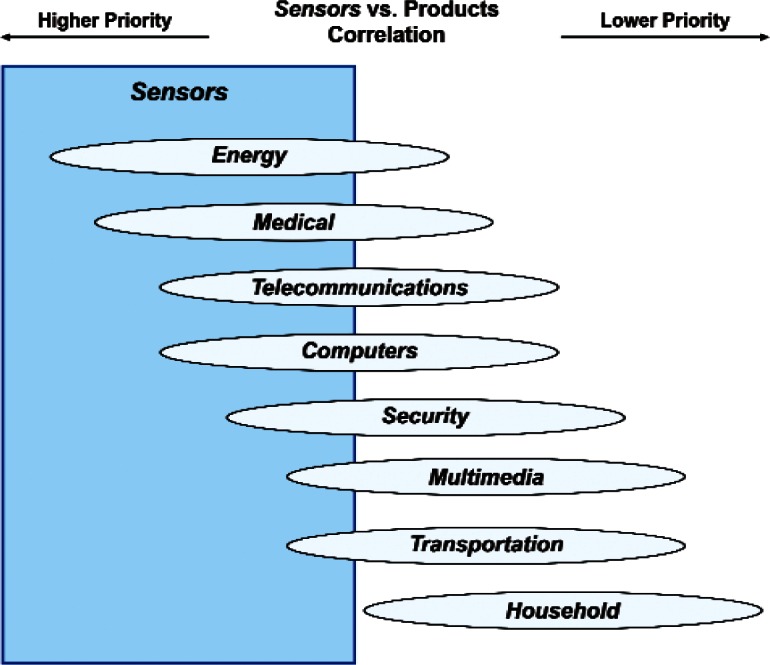
Schematic of the correlation of the relative importance of Cross-Cutting Technology item *Sensors* with the Products category.

Our general conclusions from the foregoing analyses are:

1. To increase confidence in the ranked Survey items, we may arrange them, as follows, in subgroups based on median ranks for each of the five category types.
  **Properties** (Fig. 9 and Table 4)
    Sub-Group 1 - *Electronic and Electrical*
    Sub-Group 2 - *Optical*
    Sub-Group 3 - *Biological; Chemical; Radio Frequency; and Magnetic*
  **Products** (Fig. 10 and Table 5)
    Sub-Group 1 - *Energy; Medical Products*; and *Computers*
    Sub-Group 2 - *Telecommunications*
    Sub-Group 3 - *Security and Emergency Response* and *Multimedia Consumer Electronics*
    Sub-Group 4 - *Household and Consumer Applications*
    Sub-Group 5 - *Transportation*
  **Cross-Cutting Technologies** (Fig. 11 and Table 6)
    Sub-Group 1 - *Sensors* and *Fabrication Tools*
    Sub-Group 2 - *Nano-electromechanical Systems*
    Sub-Group 3 - *Performance Assessment; Analytical Equipment; EHS; Instrumentation;* and *Optical Technologies*
  **Discipline Areas** (Fig. 12 and Table 7)
    Sub-Group 1 - *Measurement and Performance*
    Sub-Group 2 - *Design and Development; HSE*; and *Dependability and Reliability*
    Sub-Group 3 - *Electromagnetic Compatibility* and *Terminology and Symbols*
  **Stages of the Linear Economic Model** (Fig. 13 and Table 8)
    Sub-Group 1 - *Basic Technical Research* and *Technology Development*
    Sub-Group 2 - *Initial Deployment* and *Commercialization*
    Sub-Group 3 - *End-use by the Customer-Consumer* and *End-of-Life*
2. Even though the ordering of individual items may change by choice of analysis procedure, we find that the above sub-groupings of the Survey items and their ordering based on the Borda global rank in Tables 4 to 8 within a median sub-group largely reflect the consensus of the multifaceted and international nano-electrotechnologies community of stakeholders.

The raw data from the Survey presented in Tables 4 through 8 are available as Microsoft Excel files. Subject to satisfying all of the criteria given in Appendix B, other analyses and correlations than those presented in the foregoing sections may be useful. The authors welcome suggestions and possible collaborations. Interested readers should send an email to the first author at herbert.bennett@nist.gov.

## Figures and Tables

**Fig. 5:**
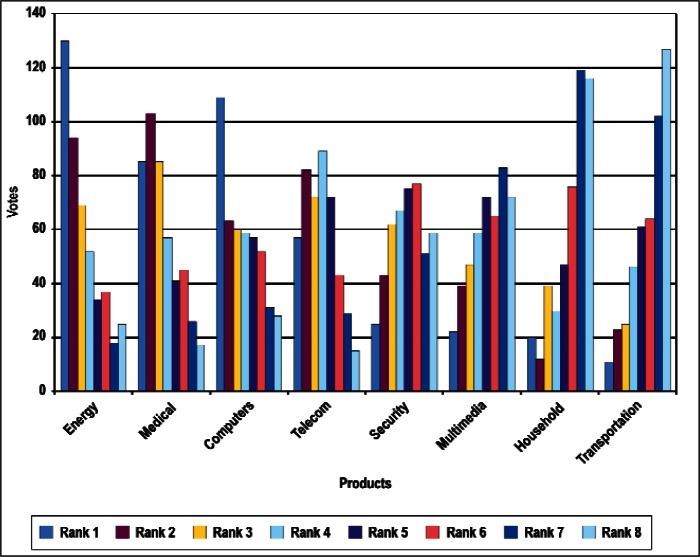
Rank Distribution of Products.

**Fig. 7:**
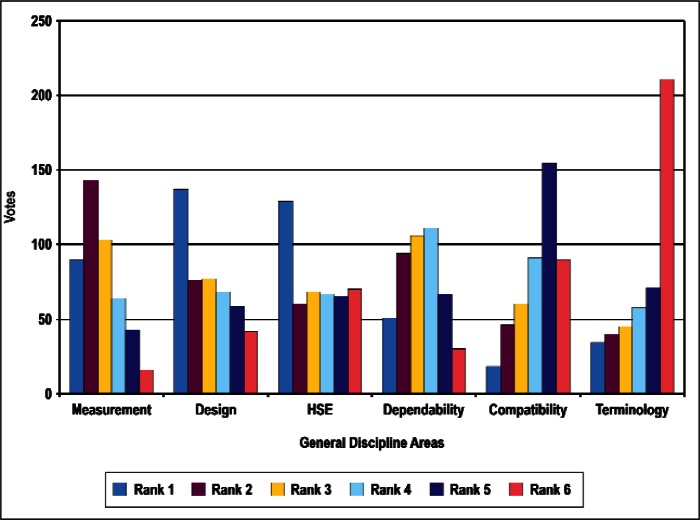
Rank Distribution of General Discipline Areas.

**Fig. 8:**
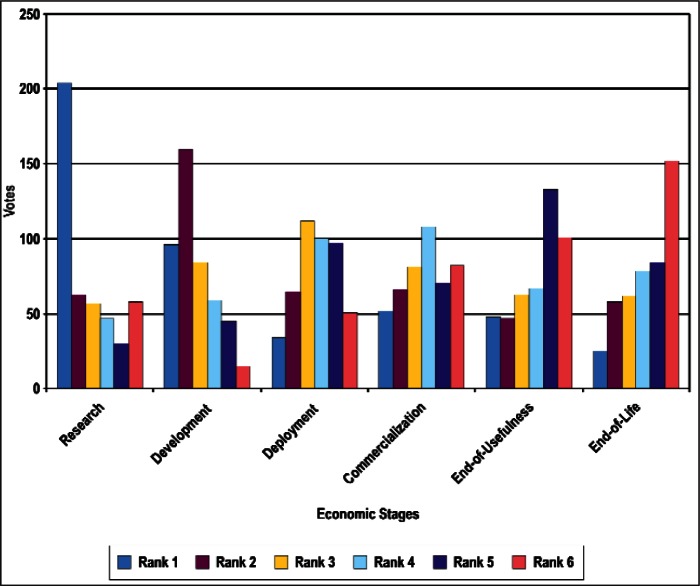
Rank Distribution of Stages of the Economic Model.

**Table 1:** Governing Principles for the Survey

I. Nano-electrotechnologies are very diverse and multi-disciplinary. IEC TC 113 members plan to use the Survey to:
• Build a consensus on key challenges to society for nano-electrotechnology implementation and inter-national markets. Possible examples include energy, healthcare, environment, emergency response, security, and multimedia communications.
• Select technologies for responding to new work items proposals on nano-electrotechnology for TC 113’s consideration.
II. At present, resources are not adequate to address simultaneously all of the fields of interest to TC 113, as cited in reference [2]. The members of the CAG decided that mechanical, physical, and thermal properties are not of primary focus in this Survey.
III. According to the IEC mission statement, the standardization efforts of TC 113 may include all electro-technologies such as electronics, magnetics and electromagnetics, electroacoustics, multimedia, telecommunication, and energy production and distribution, as well as associated general disciplines.
IV. The linear economic model for innovation in nano-electrotechnologies has six stages ranging from research and development to deployment, end use, and disposal or recycling. This linear economic model is a simplification to make analyzing the Survey responses more tractable. In practice, economic models for innovation and commercialization are very complex and non-linear with feed-back and feed-forward paths.

**Table 2:** Organizations contributing to promotion of the Survey

**Email Notifications to Members**	Institute for Electrical and Electronics Engineers (IEEE) Electron Devices Society
	IEEE Nanotechnology Council (NTC)
	IEEE-Standards Association
	International Technology Roadmap for Semiconductors (ITRS) Working Groups on Emerging Research Devices and Emerging Research Materials and Metrology
	International Standards Organization (ISO) Technical Committee 229 on Nanotechnologies
	Several ISO and IEC National Committees
**Articles/Notices Read by Members**	Semiconductor Equipment and Materials—Standards Watch, 18 June 2008 http://www.semi.org/cms/groups/public/documents/gg/p044042.htm
	Materials Research Society—Materials 360, Vol. 8, Issue 11, 19 June 2008 (1) http://www.mrs.org/s_mrs/doc.asp?CID=1926&DID=214177
	IEEE NTC—Weekly Community Updates, July 2008
	Institute of Physics—Nanotechweb, 8 August 2008 http://nanotechweb.org/cws/article/yournews/35341
	Nano Science and Technology Institute—Nano World News, 25 August 2008. http://www.nsti.org/news/item.html?id=277
**Exhibit Booths at Conferences**	NSTI NanoTech2008
	SEMICON West 2008
	ITRS Summer Conference 2008

**Table 3:** Breakdown of Completed Surveys Received by Country

{Key: (P) indicates a member of TC 113 with participant status; (O) indicates a member of TC 113 with observer status. The number in square brackets […] denotes the number of completed Surveys from that country. Countries are listed in alphabetical order in each column and from left to right.}		
Argentina (O) [2]	France (P) [12]	Mexico (O) [4]
Australia (O) [6]	Germany (P) [32]	Netherlands (O) [6]
Austria (O) [1]	Greece [2]	Poland (O) [2]
Bangladesh [1]	Hong Kong [1]	Portugal [2]
Belarus [1]	Hungary (O) [2]	Romania [1]
Belgium [7]	India (O) [18]	Russian Federation (P) [4]
Brazil (O) [4]	Indonesia (O) [1]	Singapore (P) [7]
Canada (P) [17]	Iran [2]	Spain (P) [8]
China [14]	Ireland [1]	Sweden (P) [2]
Colombia [2]	Israel [1]	Switzerland [8]
Croatia [1]	Italy (P) [22]	Taiwan [16]
Czech Republic (O) [1]	Japan (P) [31]	Thailand [3]
Egypt [2]	Korea (P) [12]	United Kingdom (P) [16]
European Union [1]	Lithuania [1]	USA (P) [174]
Finland (P) [1]	Malaysia (P) [6]	Venezuela [1]

**Table 4:** Consensus Priority Rankings for Properties

	Raw Data						Median and 95 % Cl	Borda Score	Global Consensus Rank
	Rank 1	Rank 2	Rank 3	Rank 4	Rank 5	Rank 6			
Electronic and Electrical	292	57	58	26	13	13	1 (± 0.07)	2,386	1
Optical	17	115	112	105	78	32	3 (± 0.15)	1,628	2
Biological	68	73	68	75	77	98	4 (± 0.22)	1,522	3
Chemical	37	86	70	68	113	85	4 (± 0.22)	1,447	4
Radio Frequency	34	83	69	78	63	132	4 (± 0.29)	1,387	5
Magnetic	11	45	82	107	115	99	4 (± 0.15)	1,269	6

**Table 5:** Consensus Priority Rankings for Products

	Raw Data								Median and 95 % Cl	Borda Score	Global Consensus Rank
	Rank 1	Rank 2	Rank 3	Rank 4	Rank 5	Rank 6	Rank 7	Rank 8			
Energy	130	94	69	52	34	37	18	25	3 (± 0.22)	2,680	1
Medical Products	85	103	85	57	41	45	26	17	3 (± 0.22)	2,564	2
Computers	109	63	60	59	57	52	31	28	3 (± 0.22)	2,442	3
Telecommunication	57	82	72	89	72	43	29	15	4 (± 0.22)	2,397	4
Security and Emergency Response	25	43	62	67	75	77	51	59	5 (± 0.22)	1,900	5
Multimedia Consumer Electronics	22	39	47	59	72	65	83	72	5 (± 0.22)	1,747	6
Household and Consumer Applications	20	12	39	30	47	76	119	116	7 (± 0.22)	1,398	7
Transportation	11	23	25	46	61	64	192	127	6 (± 0.22)	1,396	8

**Table 6:** Consensus Priority Rankings for Cross-Cutting Technologies

	Raw Data								Median and 95 % Cl	Borda Score	Global Consensus Rank
	Rank 1	Rank 2	Rank 3	Rank 4	Rank 5	Rank 6	Rank 7	Rank 8			
Sensors	100	94	60	49	51	45	34	26	3 (± 0.22)	2,496	1
Fabrication Tools	109	61	66	52	47	40	40	44	3 (± 0.29)	2,387	2
Nano-electromechanical Systems	59	71	59	58	65	45	46	56	4 (± 0.29)	2,156	3
Performance Assessment	55	54	58	57	57	61	60	57	5 (± 0.29)	2,039	4
Analytical Equipment	30	57	54	70	80	74	58	36	5 (± 0.22)	2,007	5
EHS	71	40	45	39	48	54	66	96	5 (± 0.29)	1,895	6
Instrumentation	13	39	58	73	60	71	84	61	5 (± 0.22)	1,772	7
Optical Technologies	22	43	59	61	51	69	71	83	5 (± 0.29)	1,772	8

**Table 7:** Consensus Priority Rankings for General Discipline Areas

	Raw Data						Median and 95 % Cl	Borda Score	Global Consensus Rank
	Rank 1	Rank 2	Rank 3	Rank 4	Rank 5	Rank 6			
Measurement and Performance	90	143	103	64	43	16	2 (± 0.15)	1,961	1
Design and Development	137	76	77	68	59	42	3 (± 0.22)	1,874	2
Health, Safety, and Environment	129	60	68	67	65	70	3 (± 0.29)	1,747	3
Dependability and Reliability	51	94	106	111	67	30	3 (± 0.15)	1,697	4
Electromagnetic Compatibility	18	46	60	91	154	90	5 (± 0.15)	1,249	5
Terminology and Symbols	34	40	45	58	71	211	5 (± 0.22)	1,111	6

**Table 8:** Consensus Priority Rankings for Economic Stages

	Raw Data						Median and 95 % Cl	Borda Score	Global Consensus Rank
	Rank 1	Rank 2	Rank 3	Rank 4	Rank 5	Rank 6			
Basic Technical Research	204	63	57	47	30	58	2 (± 0.22)	2,026	1
Technology Development	96	160	84	59	45	15	2 (± 0.15)	1,994	2
Initial Deployment	34	65	112	100	97	51	4 (± 0.15)	1,522	3
Commercialization	52	66	81	108	70	82	4 (± 0.22)	1,512	4
End-use by the Customer-Consumer	48	47	63	67	133	101	5 (± 0.15	1,343	5
End-of-Life	25	58	62	78	84	152	5 (± 0.22)	1,242	6

**Table 9:** Survey Results Relevant to a Bimodal Distribution for Crosscutting Technology: *EHS Applications and Effects*

Survey Results	Rankings
	
Rank Data—Cross-Cutting Technologies (Table 6)	Significant number of votes for both high rank and low rank (bimodal)
Priority Ranking for General Discipline Area: *Health, Safety, and Environment* (Fig. 7).	Large number of votes for rank 1; general population supports it as a priority (not bimodal)
Correlation for General Discipline Area: *Health, Safety and Environment* versus Cross-Cutting Technologies (Fig. 22)	Majority ranked *EHS Applications and Effects* first.
Correlation for Products: *Medical versus Cross-Cutting Technology* (Fig. 16)	Majority ranked *EHS Applications and Effects* first.
Correlation for Products: *Energy* versus Cross-Cutting Technology (Fig. 14)	*EHS Applications and Effects* ranked in the next to the last sub-group or tier.
Correlation for Place of Employment: *Universities* versus Cross-Cutting Technologies (Fig. 26)	*EHS Applications and Effects* ranked in the next to the last sub-group or tier.
Correlation for Products: *Computers* versus Cross-Cutting Technology (Fig. 15)	Majority ranked *EHS Applications and Effects* last.
Correlation for Products: *Telecommunication and Data Communications* versus Cross-Cutting Technology (Fig. 17)	Majority ranked *EHS Applications and Effects* last.
Correlation for General Discipline Area: *Design and Development* versus Cross-Cutting Technologies (Fig. 21)	Majority ranked *EHS Applications and Effects* last.
Correlation for General Discipline Area: *Measurement and Performance* versus Cross-cutting Technologies (Fig. 23)	Majority ranked *EHS Applications and Effects* last.
Correlation for Place of Employment: *Manufacturing Companies* versus Cross-Cutting Technologies (Fig. 27)	Majority ranked *EHS Applications and Effects* last.

## Appendix A Survey Text

**IEC TC113 NANO-ELECTROTECHNOLOGY SURVEY to establish priorities for standards development and measurements for electrical and electronic products and systems**

### About us

In 2006, the International Electrotechnical Commission (IEC) http://www.iec.ch/ established the Technical Committee 113 (TC 113) on *Nanotechnology standardization for electrical and electronic products and systems (Nano-electrotechnology)*. The TC 113 Chairman’s Advisory Group (CAG) formed an international TC 113 Survey Project Team to prepare this survey. The results from this survey will be used by the TC 113 to assist in identifying those nanotechnology areas for which standards are critically needed to accelerate innovation.

In its role to support international standards development for nano-electrotechnology, the Electronics and Electrical Engineering Laboratory (EEEL) at the U.S. National Institute of Standards and Technology (NIST) has contracted with Energetics Incorporated http://www.energetics.com/ to conduct, analyze, and report on the survey results. NIST is the national measurement institute (NMI) for the U.S. and has a strong interest in understanding measurement priorities in this field. The U.S. Government offers the following notice about surveys that it is conducting or that it is funding others to conduct:

### Paperwork Reduction Act

This survey contains collection of information requirements subject to the U.S. Paperwork Reduction Act. Notwithstanding any other provisions of the law, no person is required to respond to, nor shall any person be subject to penalty for failure to comply with, a collection of information subject to the requirements of the Paperwork Reduction Act. The estimated response time for this survey is 8 minutes. The response time includes the time for reviewing instructions, searching existing data sources, gathering and maintaining the data needed, and completing and reviewing the collection of information. Please send comments regarding this estimate or any other aspects of this collection of information, including suggestions for reducing the length of this questionnaire, to the National Institute of Standards and Technology, Herbert Bennett, TC113Survey@nist.gov. The U.S. Office of Management and Budget (OMB) number for this survey is OMB 0693-0033, expiring on 7/31/2009.

SIDEBAR every page: For more information on the conduct, design, or outcome of this survey, please contact TC 113 Survey Webmaster@energetics.com

### Goals and Objectives

Recently, the International Electrotechnical Commission (IEC) http://www.iec.ch/ established the Technical Committee 113 (TC 113) on Nanotechnology standardization for electrical and electronic products and systems (Nano-electrotechnology). The committee was created to identify and help address the future needs for standards for nanotechnology relevant to nano-electrotechnology. TC 113 has a membership of 26 countries, of which 15 are participating countries from four continents.

Due to the potentially wide application of nano-electrotechnology, the TC 113 has a need to prioritize future standardization work to make sure that the most important standards are developed first. The Technical Committee members will use this Survey to assist in identifying those nanotechnology areas relevant to electronics and electrical products for which standards are critically needed to accelerate innovation.

#### Your input is critical to the TC 113 process

Your survey responses will help prioritize the TC 113’s actions over the next few years.

The goal of this Survey is to begin building a consensus among members of the nano-electrotechnology community on a framework leading to standards development. Your responses to this survey will help the IEC TC 113 set priorities. Specifically, the TC 113 wishes to:

1. Set procedures for ranking new documents for comment (DC) and new work item proposals (NWIPs) in priority order;
2. Identify members for work groups to improve DCs and complete high priority NWIPs; and
3. Respond to DCs and NWIPs from IEC National Committees.

We invite all members of the nano-electrotechnology community to complete this Web-based survey within two weeks (DATE). This survey should take you about 8 minutes to complete.

### Governing Principles

- Nano-electrotechnologies are very diverse and multidisciplinary. IEC TC 113 members plan to use the Survey to:
  - Build a consensus on key challenges to society for nano-electrotechnology implementation and international markets. Possible examples include energy, healthcare, environment, emergency response, security, and multimedia communications.
  - Select technologies for responding to new work items proposals on nano-electrotechnology for TC113’s consideration.
- Present resources are not adequate to address simultaneously all of the fields of interest to TC 113 as cited in the May 2007 IEC E-TECH article. http://www.iec.ch/online_news/etech/arch_2007/etech_0507/spotlight.htm?mlref=etech
  Fields of interest to TC 113 cited therein are:
  - Performance and reliability assessment for nanoelectronics
  - Analytical equipment and techniques for measurement of electrotechnical properties
  - Fabrication tools for integrated circuits (electronic, photonic, and optoelectronic)
  - Nano structured sensors
  - Nano-electronics, materials and devices
  - Opto-electronics
  - Optical materials and devices
  - Organic (Opto) electronics
  - Magnetic materials and devices
  - Radio frequency devices, components and systems
  - Electrodes with nano-structured surfaces
  - Electrotechnical properties of nano-tubes/nanowires
  - Fuel cells
  - Bioelectronic applications.
- According to the IEC mission statement (http://www.iec.ch/about/mission-e.htm) the standardization efforts of TC 113 may include all electrotechnologies such as electronics, magnetics and electromagnetics, electroacoustics, multimedia, telecommunication, and energy production and distribution, as well as associated general disciplines as follows:
  - Terminology, Nomenclature, and Symbols
  - Design and Development
  - Measurement and Characterization
  - Performance Assessment
  - Dependability and Reliability
  - Electromagnetic Compatibility
  - Safety and Environment
- The linear economic model for innovation in nano-electrotechnologies has the following six stages:
  - Research
  - Development
  - Initial deployment
  - Commercialization (large-scale, high-volume manufacturing)
  - End use by the customers-consumers
  - End-of-life (disposing and recycling)

If you have comments on the completeness or relevance of principles I-IV please include them here.

### Demographics

How would you describe the nature of your work in nano-electrotechnologies?

Check only one.

1. Technical R&D ______
2. Technical Manufacturing ______
3. Management of R&D ______
4. Management of Manufacturing ______
5. Standards Developer, Administrator, or Director ______
6. Strategic Planner and Market Analyst ______
7. Other - Please be more specific ______

What is the type of institution where you are employed?

Check only one.

1. Manufacturing Company ______
2. University ______
3. Government ______
4. Trade Association ______
5. Investment Bank ______
6. Metrology Organization ______
7. Standards Developing Organization ______
8. Legal Organization ______
9. Non-Profit Organization ______
10. Research Institution ______
11. Other - Please be more specific ______

Please select your country of primary employment in the list below:

Argentina
Australia
Austria
Brazil
Canada
Czech Republic
Denmark
Finland
France
Germany
Hungary
India
Indonesia
Italy
Japan
Korea, Republic of Malaysia
Mexico
Netherlands
Poland
Portugal
Russian Federation
Singapore
Spain
Sweden
United Kingdom
United States of America

If your country of primary employment is not listed, please specify in the text box below:

{textbox}

### Nano-Electrotechnology Properties

Please rank the following nano-electrotechnology properties of concern to TC 113 in numerical priority order from 1 to 6, where 1 is most important property for TC 113 members to consider first. Please do not assign the same numerical order to more than one taxonomy category.

Priority ______ Electronic and Electrical
Priority ______ Optical
Priority ______ Magnetic
Priority ______ Radio Frequency
Priority ______ Chemical
Priority ______ Biological

### Nano-Electrotechnology Taxonomy: Products

Please rank the following TC 113 taxonomy categories in numerical priority order from 1 to 8, where 1 is most significant, i.e., the most important in terms of enabling innovations at the nanoscale. Please do not assign the same numerical order to more than one taxonomy category.

### Products

Priority ______ Computers (PDA and similar, laptop, desktop, mainframe) and Computer Peripherals (printers, monitors/displays, etc.)
Priority ______ Multimedia Consumer Electronics
Priority ______ Telecommunication and data communications (wireless and wired-physical connection)
Priority ______ Energy (Production, Conversion, and Storage)
Priority ______ Medical Products
Priority ______ Security and Emergency Response Devices and Applications
Priority ______ Household and Consumer Applications
Priority ______ Transportation (Sea/Water, Ground, Air, and Space)

Optional: Are there any other taxonomy categories not covered by the above list that would be appropriate for TC 113 to consider? If so, please cite unique categories that are not contained within the ones listed above and indicate where they rank relative to your ranking of the eight taxonomy categories listed above. For example: before 1, between 1 and 2, 2 and 3, 3 and 4…, or after 8.

{comment box}

### Nano-Electrotechnology Taxonomy: Cross-Cutting Technologies

Please rank the following TC 113 taxonomy categories in numerical priority order from 1 to 8, where 1 is most significant, i.e., the most important in terms of enabling innovations at the nanoscale. Please do not assign the same numerical order to more than one taxonomy category.

Priority______ Performance and reliability assessment for nanoelectronics
Priority ______ Analytical equipment and techniques for measurement of electro-technical properties
Priority ______ Fabrication tools for integrated circuits (electronic, photonic, optoelectronic, and mechanical)
Priority ______ Optical Technologies (Optoelectronics and Illumination)
Priority ______ Environmental, Health, and Safety (EH&S) Applications and Effects
Priority ______ Instrumentation (Test Equipment and Industrial Process Control for Use in Fabrication)
Priority ______ Nano-electromechanical systems
Priority ______ Sensors (chemical, physical, mechanical, etc.)

Optional: Are there any other taxonomy categories not covered by the above list that would be appropriate for TC 113 to consider? If so, please cite unique categories that are not contained within the ones listed above and indicate where they rank relative to your ranking of the eight taxonomy categories listed above. For example: before 1, between 1 and 2, 2 and 3, 3 and 4…, or after 8.

{comment box}

### IEC General Discipline Areas

Considering the IEC General Discipline Areas for nano-electro-technologies given in the IEC Mission Statement (Governing Assumption III), please rank them in numerical priority order from 1 to 6, where 1 is most significant for TC 113 members to consider first. Please do not assign the same numerical order to more than one focus area.

Priority ______ Terminology and Symbols
Priority ______ Design and Development
Priority ______ Measurement and Performance
Priority ______ Dependability and Reliability
Priority ______ Electromagnetic Compatibility
Priority ______ Health, Safety and Environment

### Stages of the Economic Model

Considering the stages of the economic model, please rank them in numerical priority order from 1 to 6, where 1 is most significant for TC 113 members to consider first (i.e., where standards are required). Please do not assign the same numerical order to more than one focus area.

Priority ______ Basic Technical Research
Priority ______ Technology Development (prototype development)
Priority ______ Initial Deployment
Priority ______ Commercialization (large-scale, high volume manufacturing)
Priority ______ End use by the customer-consumer
Priority ______ End-of-Life (disposing and recycling)

### Additional Comments

Optional: Please provide any additional comments concerning what you think should be the action items for the IEC TC 113 members in the near-term (1 to 3 years), mid-term (3 to 10 years), and long-term (greater than 10 years).

{comment box}

### Potential Participation on the Work of the Technical Committee—IEC TC 113

1. Would you be willing to serve as an expert contributing to the **IEC TC 113—Nanotechnology standardization for electrical and electronic products and systems on nanotechnology?**
  - If no, go straight to 2.
  - If yes, please continue:
    An IEC member is called a National Committee (NC), and each NC represents its nation's electrotechnical interests in IEC management and standardization work.
    *If you are in a country that already participates in the work of the IEC TC 113, or has Observer status, please email* the Secretary for your NC directly by clicking on the appropriate links in the TC 113 Country Table below. The country information will open in a separate window. After sending the e-mail to the Secretary of your NC, you will have to use your browser to close the page in order to return to continue the survey.
2. If your country is not listed in the above table, please e-mail the IEC TC 113 Secretary Dr. Norbert Frabricius, at Norbert.Fabricius@nanomikro.fzk.de for information to contact your National Committee or to participate as an individual expert if your country does not have an IEC National Committee.

**Table:**

Country	Country Code	P/O Status
Argentina	AR	Observer
Australia	AU	Observer
Austria	AT	Observer
Brazil	BR	Observer
Canada	CA	Participant
Czech Republic	CZ	Observer
Denmark	DK	Observer
Finland	FI	Participant
France	FR	Participant
Germany	DE	Participant
Hungary	HU	Observer
India	IN	Observer
Indonesia	ID	Observer
Italy	IT	Participant
Japan	JP	Participant
Korea, Republic of	KR	Participant
Malaysia	MY	Participant
Mexico	MX	Observer
Netherlands	NL	Observer
Poland	PL	Observer
Portugal	PT	Observer
Russian Federation	RU	Participant
Singapore	SG	Participant
Spain	ES	Participant
Sweden	SE	Participant
United Kingdom	GB	Participant
United States of America	US	Participant

We thank you for taking advantage of this unique opportunity to contribute to and harmonize nano-electro-technology standardization efforts worldwide. We will further appreciate your contributions if you volunteered to serve as an expert. Please include your e-mail address if you would like to receive an e-mail notice with a link to download a copy of the report for this survey. A copy of your responses will be e-mailed to you.

{textbox}

### Acronyms

ANSI: American National Standards Institute
DC: Documents for comment
EEEL: Electronics and Electrical Engineering Laboratory (NIST)
IEC: International Electrotechnical Commission (web link)
NC: National Committee (country IEC member)
NEMS: Nanoelectromechanical Systems
NIST: National Institute of Standards and Technology
NWIP: New Work Item Proposal (proposal for the preparation of a standard or a series of related standards in the field covered by an existing technical committee of ISO or IEC. The proposer of the NWIP is a national committee, for the US it is ANSI.)
TC 113: Nanotechnology standardization for electrical and electronic products and systems (IEC TC 113)

We thank you for completing this Survey.

## Appendix B Statistical Formulas and Quartiles and Medians

This first part of this Appendix is based on generalizing the equations in Appendix A of reference 7 for the cases in this Survey. The second part of this Appendix is based on documenting how the software that we use computes medians and quartiles.

### Part 1 – Statistical Formulas

We treat the ranks as an ordinal variable and use the median as an estimate of the central tendency [8]. The 95 % confidence interval for *r_m_* is [*r*_lower_, *r*_upper_] defined as

$$\begin{array}{l} \Delta m:=1.57(r_{3}-r_{1})/\sqrt{N}\\ r_{upper}:=\min\{r_{m}+\Delta m,r_{3}\}\\ r_{lower}:=\max\{r_{m}-\Delta m,r_{1}\} \end{array}$$ (B.1)

where *r_m_* is the median rank, *r*_3_ and *r*_1_ are the 3*rd* and 1*st* quartile ranks, and *N* is the number of respondents. In other words, the confidence interval is symmetric about the median. When the interval extends beyond the quartile, we use the interval value and not the quartile value in the Figures.

We follow Lehmann [9] for computing the Friedman’s statistic. Because the Survey has *n_i_* items for each category type *i* (i.e., “treatments”) and repeat rankings are not allowed, if one assumes *H*_0_ is true, then the mean item rank is (*n_i_* − 1)/2. Friedman’s statistic is the scaled sum of squared differences,

$$Q=\frac{12N}{n_{i}(n_{i}+1)}\sum_{s=1}^{n_{i}}{\left(\bar{R_{s}}-[(n_{i}+1)/2]\right)}^{2}.$$ (B.2)

Here *N* is the number of respondents and $\bar{R_{s}}$ is the mean of the *s*-th item. We reject *H*_0_ for large values of *Q*. Under the normalization (B.2), the large *N* asymptotic distribution for *Q* is a chi-square variate with d = (*n_i_* – 1) degrees of freedom, *χ*^2^*_d_*. In this paper, we consider only those subcategories of respondents for which *N* is sufficiently large that this asymptotic distribution is valid [12].

We compute confidence *p*-values as follows. In place of *Q*, for consistency across different size groups, we report Kendall’s *W*,

$$W:=Q/N(n_{i}-1).$$ (B.3)

This rescaling of *Q* is such that 0 ≤ *W* ≤ 1. Kendall and Smith [13] provide other interpretations of *W*.

As an example, using the data of Table 6, we compute *Q_all_* = 182.41 and the associated *W_all_* = 0.0568 (*N* = 459 for all survey respondents). Using the complementary cumulative distribution function of a *χ*^2^*_d_* random variable, the probability of observing *Q* ≥ *Q_all_* when *H*_0_ is true is computed by,

$$p_{all}=1-F_{\chi_{d}^{2}}(Q_{all})=0.$$ (B.4)

In this example *Q_all_* = 182.41 is sufficiently large that *p_all_* is effectively zero. Because the probability of observing *Q_all_* (or higher) when *H*_0_ is true is extremely small, we may then assert that *H*_0_ is false.

### Part 2 – Quartiles and Median

The Survey software (SelectSurvey.NET 2.8.7) produces an Excel file that contains the raw data for the 459 completed responses. This file also can be used for input into Minitab. We use Excel in Microsoft Office 2003 SP3 to compute Friedman’s statistic *Q*, Kendall’s *W*, quartiles, and medians. We use Minitab Release 14.1 to compute confidence *p*-values and to verify the Friedman’s statistic *Q* from Excel.

#### Minitab

##### Quartiles

In Minitab (http://www.minitab.com/), after the data is arranged in ascending order, the first (Q_1_) and third (Q_3_) quartiles are determined by the following equations:

$$\begin{array}{l} Q_{1}=(n+1)/4,\\ Q_{3}=3(n+1)/4, \end{array}$$ (B.5)

where *n* is the number of observations in the data set. For example, in a data set with 184 observations, Q_1_ = (184 + 1)/4 = 46.25. Since Q_1_ is not an integer, interpolation is used to determine the value y_Q1_ for the first quartile using the 46th and 47th observations in the ordered data set. If Q_1_ had been an integer, y_Q1_ would be the value associated with the Q_1_. In the data set of this example, the values in the 46th and 47th observations are 2 and 3, respectively. Through interpolation, the value that Minitab produces for the first quartile is 2.25. The interpolation is as follows:

$$y_{Q1}=y_{0}+(x–x_{0})[(y_{1}–y_{0})/(x_{1}–x_{0})],$$

where,

y_Q1_ = value to be determined,
y_0_ = value in the 46th observation = 2,
y_1_ = value in the 47th observation = 3,
x = Q_1_ = 46.25,
x_0_ = integer observation below Q_1_ = 46,
x_1_ = integer observation above Q_1_ = 47

Substituting the values in the above ordered data set give:

$$y_{Q1}=2+(46.25–46)*[(3–2)/(47–46)]=2+0.25\times 1=2.25.$$

##### Median

In Minitab, if *n* is odd, the median is the value in the middle of a data set organized in ascending order. If *n* is even, the median is the average of the two middle values. For a data set where *n* = 184 and the two values in the middle, the 92nd and 93rd observations, are 4 and 5 respectively, Minitab averages these two values to produce a median of 4.5.

#### Excel

Excel determines Q_1_, Q_3_, and the median in a somewhat different manner than Minitab, which may produce different results.

*Quartiles*: With the data arranged in ascending order, Excel computes the quartiles by the following equations:

$$\begin{array}{l} Q_{1}=(n+3)/4,\\ Q_{3}=(3n+1)/4. \end{array}$$ (B.6)

Thus, using the above example where *n* = 184 gives Q_1_ = (184 + 3)/4 = 46.75. Interpolation is still used to determine the quartile values when the resulting observation is not an integer. Therefore, using the foregoing data set, Excel produces the following result for y_Q1_:

$$y_{Q1}=2+(46.75–46)*[(3–2)/(47–46)]=2+0.75\times 1=2.75.$$

This value is different from Minitab’s 2.25 for y_Q1_.

##### Median

In Excel, the middle position Q_2_ is determined by following equation:

$$Q_{2}=(n+1)/2.$$

Therefore, when n = 184, Q_2_ = (184 + 1)/2 = 92.5. As with the first and third quartiles, Excel interpolates for the median value when the resulting observation is not an integer. Using the foregoing data set gives:

$$y_{Q2}=4+(92.5–92)*\left[\left(5–4)/(93–92\right)\right]=4+0.5\times 1=4.5.$$

This value is the same as that produced by Minitab for the median value.
